# Healthcare-Associated Infections After Aneurysmal Subarachnoid Hemorrhage: A Retrospective Single-Center Cohort Study

**DOI:** 10.3390/jcm15176718

**Published:** 2026-08-29

**Authors:** Aleksandra Kosikowska, Aleksandra Tołkacz, Zuzanna Nowak, Adrianna Lebiedzińska, Jarosław Kędziora, Waldemar Goździk, Jowita Woźniak, Małgorzata Burzyńska

**Affiliations:** 1 Scientific Student Club, Department of Anesthesiology and Intensive Care, University Clinical Hospital in Wroclaw, 50-556 Wroclaw, Poland; 2 Clinical Department of Anesthesiology and Intensive Therapy, University Clinical Hospital in Wroclaw, 50-556 Wroclaw, Poland; 3 Clinical Department of Anesthesiology and Intensive Therapy, Faculty of Medicine, Wroclaw Medical University, 50-367 Wroclaw, Poland; 4 Clinical Department of Neurosurgery, University Centre for Neurology and Neurosurgery, University Clinical Hospital in Wroclaw, 50-556 Wroclaw, Poland

**Keywords:** aneurysmal subarachnoid hemorrhage, cross-infection, intensive care units, neurocritical care, in-hospital mortality, treatment outcome

## Abstract

**Background/Objectives:** Healthcare-associated infections (HAIs) frequently complicate aneurysmal subarachnoid hemorrhage (aSAH) requiring neurocritical care. We assessed their incidence, timing, microbiology, associated factors, and outcomes. **Methods:** This retrospective, single-center cohort study included 106 consecutive adults with acute aSAH admitted to a neurocritical care unit during 2019–2024. Time to first HAI was analyzed using cause-specific Cox regression with competing risks; to limit immortal-time bias, HAI was modeled as a time-dependent exposure in outcome analyses. **Results:** HAIs occurred in 47 patients (44.3%; 95% CI 35.2–53.8); median onset was 10 days (IQR 8–12). Seventy-six episodes were recorded; site-specific figures denote affected patients, with no recurrent same-site episodes: ventilator-associated pneumonia, 30 (28.3%); catheter-associated urinary tract infection, 24 (22.6%); cerebrospinal fluid infections, 13 (12.3%); and central line-associated bloodstream infection, 9 (8.5%). *Acinetobacter baumannii* predominated, accounting for all extensively drug-resistant isolates. High World Federation of Neurosurgical Societies grade was associated with the first HAI (HR 4.168; 95% CI 2.072–8.384), as was higher modified Fisher grade in sensitivity analysis (HR 2.886; 95% CI 1.237–6.735). Time-dependent HAI was associated with a lower hazard of live discharge (HR 0.295; 95% CI 0.164–0.529) but not with in-hospital mortality (HR 1.178; 95% CI 0.478–2.902). Its association with the discharge Glasgow Outcome Scale (GOS) was threshold-dependent (GOS ≤ 1: OR 0.715, 95% CI 0.264–1.933; GOS ≤ 3: OR 7.939, 95% CI 2.580–24.436). **Conclusions:** HAIs affected nearly half of this cohort. Greater initial severity was associated with a higher hazard of first HAI; HAI, in turn, was associated with prolonged hospitalization and unfavorable functional outcome, but not with mortality. These associations are observational, not causal. Surveillance, timely diagnosis, and infection prevention remain integral to neurocritical care in aSAH.

## 1. Introduction

Aneurysmal subarachnoid hemorrhage (aSAH) is a severe form of hemorrhagic stroke associated with substantial early mortality and long-term neurological disability [1,2]. Approximately 85% of spontaneous subarachnoid hemorrhages result from the rupture of an intracranial aneurysm and are therefore classified as aneurysmal subarachnoid hemorrhage (aSAH) [3]. Although it accounts for only 2–5% of all strokes, aSAH is associated with substantial mortality and long-term disability. Despite advances in neurocritical care and aneurysm treatment, up to 30% of patients die within 90 days, and many survivors experience persistent neurological deficits [4,5].

The clinical course after aneurysm rupture is determined by an interplay between early brain injury (EBI) and subsequent neurological and systemic complications. EBI involves blood–brain barrier disruption, neuroinflammation, endothelial dysfunction, and activation of the sympathetic nervous system, and may contribute to the development of delayed cerebral ischemia (DCI), cerebral vasospasm, hydrocephalus, and cerebral edema [3,4,5]. At the same time, acute brain injury can induce systemic, neuroendocrine, and immune alterations. Activation of the sympathetic nervous system and the hypothalamic–pituitary–adrenal (HPA) axis following aneurysm rupture may represent early mechanisms linking central nervous system injury to systemic immune responses [6,7]. This initial inflammatory response may subsequently be accompanied by a state of immune dysfunction or immunosuppression, potentially increasing susceptibility to infectious complications, particularly pneumonia in patients requiring mechanical ventilation [8,9].

Patients with aSAH frequently require prolonged neurocritical care and may undergo mechanical ventilation, invasive neuromonitoring, urinary catheterization, central venous access, and external cerebrospinal fluid drainage. Consequently, they are particularly susceptible to healthcare-associated infections (HAIs), most commonly ventilator-associated pneumonia (VAP), catheter-associated urinary tract infections (CAUTI), central line-associated bloodstream infections (CLABSI), and cerebrospinal fluid infections [10,11].

Infection may also act as an additional “second hit” to the vulnerable brain by amplifying systemic and intracranial inflammation, endothelial dysfunction, impaired autoregulation and microcirculation, blood–brain barrier disruption, and microthrombosis [12,13,14]. These mechanisms provide a biological rationale for a possible association between infection and neurological complications such as DCI. However, the present study did not directly investigate these pathophysiological mechanisms, and any potential link between infection and secondary brain injury should therefore be interpreted cautiously. Previous observational studies have suggested potential associations among infection or sepsis, DCI, and unfavorable functional outcomes, but the available evidence remains limited and inconsistent [15,16,17,18]. Importantly, the temporal relationship between these complications may substantially influence their interpretation. For example, Centner et al. reported that the association between sepsis and DCI was attenuated when the temporal sequence of the two events was considered [16]. Thus, infection may contribute to secondary neurological deterioration, but it may also represent a marker of greater initial disease severity or prolonged critical illness. Conversely, more severe EBI may predispose patients to both DCI and infection. The relationship between infection, disease severity, and neurological outcome should therefore not be assumed to be unidirectional.

Despite the clinical relevance of HAIs after aSAH, several important aspects of their epidemiology and clinical significance remain insufficiently characterized. In particular, the timing of the first HAI in relation to the early and subsequent clinical course after aneurysmal rupture has not been adequately described; it also remains unclear whether infections develop predominantly during the early phase after hemorrhage or later during prolonged neurocritical care. Local microbiological patterns, antimicrobial resistance profiles, and device-associated infection rates are also relevant for guiding preventive and empirical antimicrobial treatment and infection prevention strategies. In addition, the association between HAI and clinical outcomes is difficult to interpret because infection is an inherently time-dependent event and may reflect and potentially contribute to greater disease severity and prolonged exposure to invasive devices.

The primary aim of this study was to characterize the occurrence, type, microbiological profile, and timing of the first HAI in patients with aSAH treated in the neurointensive care unit and to identify baseline and early clinical factors associated with its development. Device-associated infection rates and antimicrobial resistance patterns were additionally evaluated as descriptive measures of the local epidemiology of HAIs. We hypothesized that greater initial neurological severity and the early requirements for invasive organ support would be associated with a higher risk of developing the first HAI.

A secondary, exploratory aim was to evaluate the associations between HAI occurrence and DCI, transcranial Doppler (TCD)-detected cerebral vasospasm, duration of mechanical ventilation, exposure to invasive devices, ICU and hospital length of stay, in-hospital mortality, and functional status at hospital discharge. Given the observational design and the time-dependent nature of HAI occurrence, these analyses were considered exploratory and were interpreted as associations rather than as evidence of independent causal effects.

## 2. Materials and Methods

### 2.1. Study Design and Patient Selection

A retrospective, single-center cohort study was conducted among consecutive adult patients with acute aSAH admitted to the Intensive Care Unit of the University Clinical Hospital in Wroclaw between January 2019 and December 2024. A total of 140 patients were initially assessed for eligibility.

Of these, 106 patients (57 women and 49 men) met the inclusion criteria and were included in the final analysis. Eligible patients were ≥18 years of age and had experienced their first-ever episode of aSAH secondary to the rupture of an intracranial aneurysm. The diagnosis was confirmed by non-contrast cranial computed tomography (CT), CT angiography (CTA), and/or digital subtraction angiography (DSA). Additional inclusion criteria were hospital admission within 24 h of symptom onset and definitive aneurysm occlusion within 48 h by microsurgical clipping or endovascular treatment.

**Figure 1:**
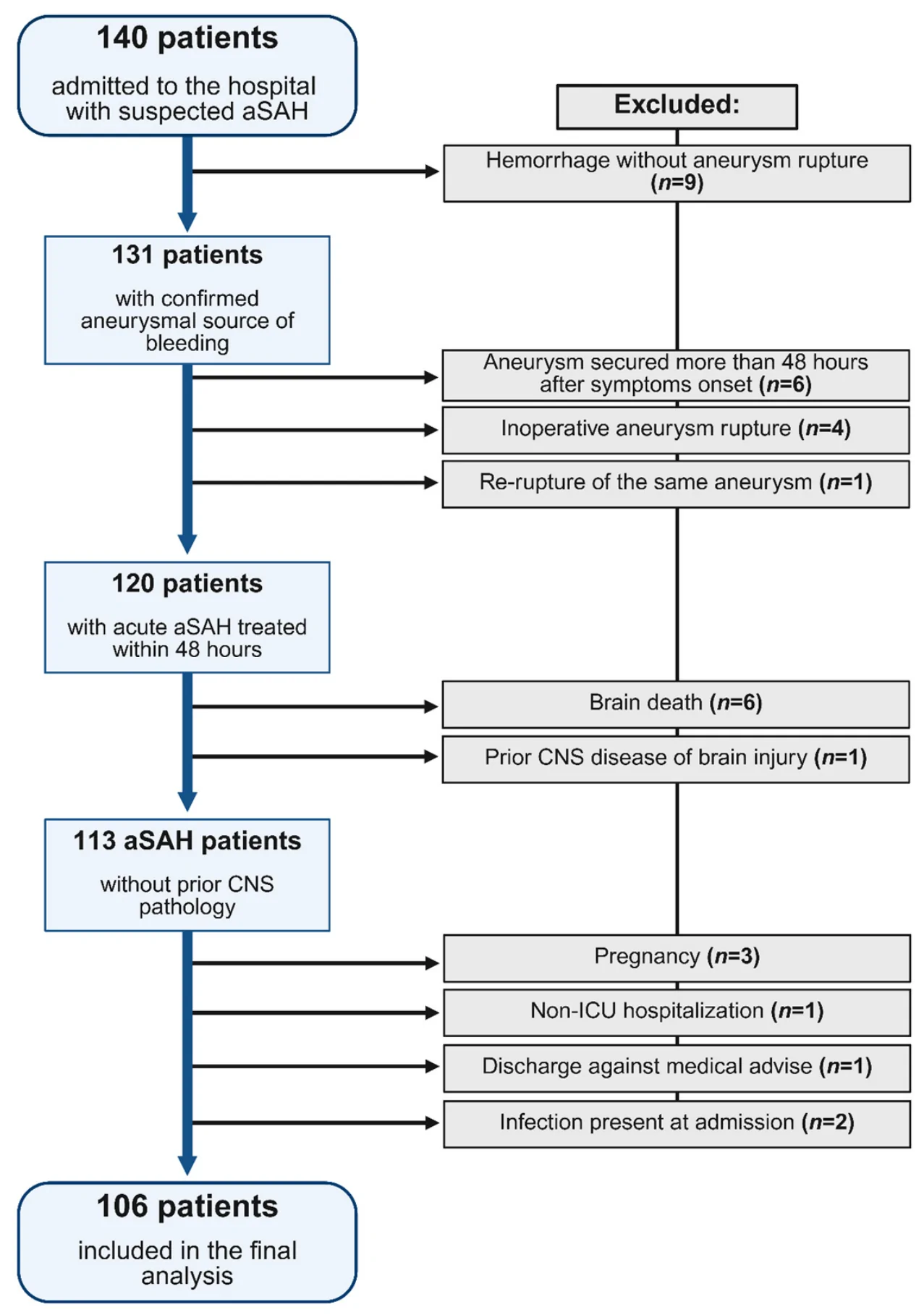
Flow diagram of patient selection for the final analysis. Abbreviations: aSAH—aneurysmal subarachnoid hemorrhage; CNS—central nervous system; ICU—intensive care unit. Created in BioRender. Burzyńska, M. (2026) https://BioRender.com/69kzweb.

Exclusion criteria included the absence of an identifiable aneurysmal source of hemorrhage, brain death, a history of brain injury or other central nervous system (CNS) disease, evidence of active infection upon admission, pregnancy, discharge against medical advice, and treatment outside the ICU. Patients with intraoperative aneurysm rupture, re-rupture of the index aneurysm, or aneurysm occlusion performed more than 48 h after symptom onset were also excluded. The patient selection process is illustrated in Figure 1.

### 2.2. Clinical Management and Data Collection

Clinical and demographic data collected for analysis included age, sex, body mass index (BMI), and chronic comorbidities such as hypertension, ischemic heart disease, diabetes mellitus, and thyroid dysfunction. Neurological status at admission was assessed using the Glasgow Coma Scale (GCS) and the Full Outline of UnResponsiveness (FOUR) score. The FOUR score evaluates eye response, motor response, brainstem reflexes, and respiratory pattern (range 0–16) [19,20]. Hemorrhage severity was assessed using the modified Fisher scale and the World Federation of Neurosurgical Societies (WFNS) grading system. Overall physiological severity was assessed using the Acute Physiology and Chronic Health Evaluation II (APACHE II) score, calculated within the first 24 h after admission.

Patients were managed according to standardized institutional neurocritical care protocols, which were initially based on contemporary guidelines and later updated to incorporate the 2023 American Heart Association/American Stroke Association (AHA/ASA) guidelines for the management of patients with aneurysmal subarachnoid hemorrhage [21,22].

The institutional policy was to secure the ruptured aneurysm as early as clinically feasible. Treatment modality was selected according to the patient’s clinical condition, hemorrhage severity, and aneurysm characteristics, including size, morphology, and location. Treatment decisions were made by a multidisciplinary team comprising neurosurgeons, interventional neuroradiologists, and neurointensivists. Acute symptomatic hydrocephalus was treated with external ventricular drainage according to clinical and radiological indications. During the later part of the study period (2023–2024), lumbar cerebrospinal fluid drainage was used more frequently. In selected patients without hydrocephalus, continuous lumbar drainage was performed according to the general principles of the EARLYDRAIN protocol, with the aim of facilitating the clearance of subarachnoid blood and its degradation products and potentially reducing the risk of delayed cerebral ischemia (DCI) [23,24,25,26,27,28,29].

All patients admitted to the neurocritical care unit underwent close neurological and systemic monitoring. Routine management included follow-up head computed tomography (CT), daily neurological assessment, continuous cardiovascular and respiratory monitoring, and surveillance for infectious complications. Daily transcranial Doppler (TCD) ultrasonography was performed throughout the period of risk for cerebral vasospasm in all patients. Vasospasm on TCD was defined as a mean middle cerebral artery blood flow velocity > 120 cm/s or an approximately 50% increase compared with the previous examination [30]. Increased cerebral blood-flow velocities suggested vasospasm; CT angiography (CTA) was performed to confirm the diagnosis and guide treatment selection [31].

Delayed cerebral ischemia (DCI) was defined according to the multidisciplinary consensus criteria proposed by Vergouwen et al. [32]. Clinical DCI was diagnosed when a patient developed a new focal neurological deficit or a decrease of at least 2 points on the Glasgow Coma Scale lasting for at least 1 h that was not attributable to other causes.

Cerebral infarction attributable to DCI was defined as a new infarction identified on CT or MRI before hospital discharge that was not present on imaging performed 24–48 h after aneurysm occlusion and could not be attributed to aneurysm treatment, ventricular catheter placement, an intraparenchymal hematoma, or another identifiable cause [32].

Nimodipine was administered routinely. Hemodynamic management was directed toward maintaining euvolemia and an arterial blood pressure appropriate for the individual patient’s clinical condition. In patients with symptomatic DCI, hemodynamic augmentation with induced arterial hypertension was used when clinically appropriate while maintaining euvolemia. Patients with persistent or severe symptomatic vasospasm despite medical treatment were considered for endovascular rescue therapy, including balloon angioplasty and/or intra-arterial administration of vasodilators. In patients with poor neurological status, hemodynamic instability, or suspected cardiac dysfunction, advanced hemodynamic monitoring was used when clinically indicated to guide assessment of cardiac output, preload, and fluid responsiveness.

Additional pharmacological and supportive therapies were provided according to institutional protocols and individual clinical needs. These included stress ulcer prophylaxis, treatment of seizures, correction of electrolyte disturbances, sedation, analgesia, venous thromboembolism prophylaxis, and early enteral nutrition whenever clinically feasible. Nutritional risk was assessed using the Nutritional Risk Screening 2002 (NRS-2002), with a score ≥ 3 indicating nutritional risk [33]. In intubated and mechanically ventilated patients, the depth of sedation and level of agitation were assessed using the Richmond Agitation–Sedation Scale (RASS), ranging from +4 (combative) to −5 (unarousable), with a score of 0 indicating an alert and calm patient [34].

### 2.3. Microbiological Surveillance and Assessment of Healthcare-Associated Infections

Routine microbiological surveillance cultures of blood, urine, and throat specimens were obtained on admission and repeated weekly during the ICU stay, in accordance with institutional practice. Additional microbiological samples were collected whenever infection was clinically suspected. Surveillance cultures were distinguished from diagnostic cultures obtained in response to clinical suspicion of infection.

Hospital records were retrospectively reviewed to determine the timing, anatomical site, and microbiological etiology of HAIs. The date of HAI onset was defined as the date on which the diagnostic criteria for the respective infection were first fulfilled. For patients who developed more than one HAI, the first documented HAI was used for the primary time-to-event analysis. Subsequent infections were recorded separately for descriptive analyses. Laboratory parameters included leukocyte count, C-reactive protein (CRP), procalcitonin (PCT), and arterial blood lactate concentrations. Chest radiography and other imaging studies were reviewed whenever clinically indicated.

Throughout the study period (2019–2024), HAI prevention was based on standardized institutional infection prevention bundles developed in accordance with evolving European Centre for Disease Prevention and Control (ECDC) criteria, recommendations from the European Network for ICU-Related Respiratory Infections (ENIRRIs) project, neurocritical care recommendations, and local microbiological surveillance data [35,36,37,38]. The bundles were incorporated into routine ICU practice and were periodically reviewed and updated in response to evolving recommendations, local microbiological epidemiology, and institutional infection surveillance data. The prevention bundles addressed the major device-associated and healthcare-associated infections encountered in the neurocritical care setting.

#### 2.3.1. Prevention of Hospital-Acquired and Ventilator-Associated Pneumonia

Measures aimed at preventing hospital-acquired pneumonia (HAP), including ventilator-associated pneumonia (VAP), comprised strict hand hygiene, elevation of the head of the bed to 30–45° when clinically feasible, aspiration prevention measures, regular oral care including toothbrushing, minimization of unnecessary sedation, daily assessment of readiness for ventilator liberation, and early mobilization whenever clinically feasible. Routine scheduled replacement of ventilator circuits was not performed.

#### 2.3.2. Prevention of Bloodstream Infections

Measures to prevent bloodstream infections (BSIs) included hand hygiene, strict aseptic technique, maximal sterile barrier precautions during central venous catheter (CVC) insertion, skin antisepsis with an alcohol-based chlorhexidine preparation, sterile dressing of the insertion site, disinfection of catheter hubs and access ports before manipulation, regular reassessment of the need for central venous access, and prompt catheter removal when no longer required. Routine scheduled CVC replacement solely for infection prevention was not performed.

#### 2.3.3. Prevention of External CSF Drain-Associated Infections

External ventricular drains (EVDs) were inserted in the operating room, whereas lumbar drains (LDs) were inserted in the ICU procedure room under sterile conditions. Both systems were maintained as closed drainage systems, with manipulation minimized and cerebrospinal fluid sampling performed aseptically only when infection was clinically suspected. Routine scheduled replacement of EVDs or LDs was not performed. Drain replacement was limited to cases of suspected or confirmed infection, obstruction or malfunction, mechanical damage, accidental disconnection, or contamination of the drainage system. The indication for continued drainage was reviewed daily.

#### 2.3.4. Prevention of Urinary Tract Infections

Urinary catheters were inserted using aseptic technique and sterile equipment only when clinically indicated. Closed drainage systems were maintained, unobstructed urine flow was ensured, unnecessary disconnections were avoided, and drainage bags were kept below bladder level. The need for continued catheterization was reassessed regularly, and catheters were removed as soon as clinically feasible. Routine catheter replacement, bladder irrigation, and prophylactic antimicrobial therapy solely for CAUTI prevention were not performed.

All patients treated in the neurocritical care unit had both a central venous catheter and an indwelling urinary catheter in place from ICU admission, in accordance with standard institutional practice for patients with aSAH requiring neurocritical care. Central venous access was used for the administration of vasoactive agents, hyperosmolar therapy, nimodipine, and intravenous fluids, whereas urinary catheterization enabled close monitoring of fluid balance and hourly urine output.

Device exposure was therefore universal in this cohort. All UTIs and BSIs classified as device-associated occurred in patients with the corresponding device in place and fulfilled the predefined criteria.

CVCs were generally maintained as long as clinically indicated because patients at risk of cerebral vasospasm and DCI could require continuous vasoactive therapy and hemodynamic augmentation. The need for central venous access was reassessed daily, and the catheter was removed once central access was no longer clinically necessary.

Compliance with infection prevention bundle components and device-associated infection rates were monitored as part of routine institutional infection control surveillance throughout the study period.

### 2.4. Empirical Antimicrobial Therapy

Empirical antimicrobial therapy was initiated when infection was clinically suspected. The selection of empirical treatment was based on the presumed anatomical site of infection, previous microbiological findings, individual patient risk factors for antimicrobial resistance, and local antimicrobial susceptibility patterns. Whenever clinically feasible, appropriate specimens were obtained before antimicrobial therapy was initiated, including blood, respiratory secretions, urine, or CSF according to the suspected source of infection. Molecular diagnostic methods were used as adjunctive tests when clinically indicated to facilitate rapid pathogen identification.

Antimicrobial therapy was subsequently modified according to pathogen identification, antimicrobial susceptibility testing, clinical response, and consultation with the clinical microbiology team. Therapy was escalated when clinically indicated and de-escalated when microbiological results allowed for the narrowing of antimicrobial coverage. Treatment duration was individualized according to the site and severity of the infection, the causative pathogen, and the clinical course.

Standard perioperative antimicrobial prophylaxis consisted primarily of intravenous cefazolin (Biofazolin; Zakłady Farmaceutyczne POLPHARMA S.A., Starogard Gdański, Poland), according to institutional practice. During 2019–2020, selected patients requiring external CSF drainage additionally received a short course of intravenous ceftriaxone in accordance with the local infection prevention protocol in place at that time. Cefazolin subsequently remained the standard perioperative prophylactic agent.

The institutional antimicrobial stewardship program included adherence to established ICU antimicrobial treatment protocols, prospective review of ongoing antimicrobial therapy, microbiological consultation, restrictions on the use of selected antimicrobial agents, de-escalation following the availability of microbiological results, and therapeutic drug monitoring of selected antimicrobial agents.

### 2.5. Definitions of Infectious Complications

#### 2.5.1. Hospital-Acquired Pneumonia (HAP) and Ventilator-Associated Pneumonia

Hospital-acquired pneumonia (HAP) was defined as pneumonia developing ≥48 h after hospital admission. Pneumonia developing ≥48 h after ICU admission was considered ICU-acquired. Ventilator-associated pneumonia (VAP) was defined as hospital-acquired pneumonia occurring in a patient in whom invasive mechanical ventilation was in place at any point during the 48 h preceding the onset of infection.

The diagnosis was based on compatible radiological, systemic, and respiratory findings, with microbiological confirmation obtained whenever available. In cases without microbiological confirmation, pneumonia was classified according to the applicable ECDC PN5 criteria [39,40]. The diagnostic assessment included radiological evidence of pneumonia on chest radiography or computed tomography, at least one systemic criterion—fever > 38 °C without another recognized cause, leukopenia < 4000 WBC/mm^3^, or leukocytosis ≥ 12,000 WBC/mm^3^—and at least two respiratory manifestations such as new-onset purulent respiratory secretions or a change in their character, cough, dyspnea or tachypnea, suggestive auscultatory findings, or worsening gas exchange with oxygen desaturation, increased oxygen requirements, or increased ventilatory support. PN5 was assigned when no positive microbiological result was available.

#### 2.5.2. Urinary Tract Infection

UTI was defined according to the ECDC HAI-Net ICU protocol, version 2.2 [39], and was classified as catheter-associated (CAUTI) when an indwelling urinary catheter had been in place at any point during the seven days preceding onset. Microbiologically confirmed symptomatic UTI required at least one compatible clinical sign or symptom together with a positive urine culture yielding ≥10^5^ microorganisms per mL, with no more than two species isolated, and no more likely alternative source of infection. Qualifying microorganisms were not restricted to bacteria. Cultures reported as mixed flora were not considered to meet the microbiological criteria for CAUTI.

Although lower quantitative thresholds of ≥10^3^ microorganisms per milliliter may be clinically relevant in symptomatic catheterized patients, the threshold of ≥10^5^ microorganisms per milliliter was applied for study classification and epidemiological surveillance. Asymptomatic bacteriuria and asymptomatic candiduria were not classified as infection [39].

#### 2.5.3. Cerebrospinal Fluid Infection

CSF infection, including meningitis or ventriculitis, was defined according to the ECDC HAI-Net ICU protocol, version 2.2 [39]. A positive CSF culture obtained for clinical diagnostic purposes fulfilled the microbiological criterion. In patients without microbiological confirmation, the diagnosis required compatible clinical findings together with CSF abnormalities suggestive of infection, including a CSF leukocyte count >5 cells/µL, an elevated protein concentration > 45 mg/dL (0.45 g/L), and a reduced glucose concentration < 40 mg/dL (2.2 mmol/L) or a CSF-to-serum glucose ratio ≤ 0.4 [41,42]. These parameters were interpreted in the clinical context and were not considered diagnostic in isolation, particularly in patients with aSAH and external CSF drainage, as subarachnoid blood, intraventricular hemorrhage, neurosurgical procedures, and CSF drainage may independently alter CSF cellular and biochemical parameters [36].

#### 2.5.4. Bloodstream Infection and Central Line-Associated Bloodstream Infection

Bloodstream infection (BSI) was defined as the isolation of a recognized pathogenic microorganism from a blood culture obtained for clinical diagnostic purposes or, in the case of common skin contaminants, the isolation of the same organism from at least two separately collected blood culture specimens in the presence of compatible clinical features such as fever, chills, or hypotension. Central line-associated bloodstream infection (CLABSI) was defined as a primary BSI occurring in a patient in whom a central venous catheter had been in place at any point during the 48 h preceding infection onset, and for whom no more likely alternative source was identified. Where available, microbiological investigations supporting catheter-related origin were recorded separately. A positive catheter culture (≥10^3^ CFU/mL by quantitative culture or >15 CFU by semiquantitative culture), a differential time to positivity of ≥2 h between central and peripheral blood cultures, or the isolation of the same microorganism from blood and catheter cultures was recorded as supporting evidence of a catheter-related origin but was not required for classification [39].

Device association was adjudicated for every episode against the recorded device exposure period, applying a single prespecified rule in accordance with the ECDC HAI-Net ICU protocol, version 2.2 [39]: an infection was classified as device-associated when the relevant device was in place at any point during the 48 h preceding the onset of infection. For urinary catheter-associated infection, the corresponding window defined in the protocol was seven days preceding onset. Where a device had been removed and subsequently reinserted, each period of device exposure was recorded separately, and device association was determined with reference to the most recent period of exposure preceding the infection. On this basis, all pneumonia episodes fulfilled the criteria for ventilator-associated pneumonia, and all bloodstream infection episodes fulfilled the criteria for central line-associated bloodstream infection. All urinary tract infections occurred while the urinary catheter was in situ.

### 2.6. Epidemiological Measures of Healthcare-Associated Infections

The frequency of each HAI category was calculated as the number of infection episodes divided by the total number of patients included in the study and expressed as a percentage.

Overall HAI density was calculated as the number of infection episodes divided by the total number of patient-days and expressed per 1000 patient-days. Two denominators were applied: total hospital patient-days, defined as the sum of days from hospital admission to discharge or death across all patients, and total ICU patient-days, defined analogously for the period of neurocritical care.

For device-associated infections, incidence density was calculated using the corresponding device-days of exposure as the denominator. VAP incidence density was expressed as the number of VAP episodes per 1000 mechanical ventilation-days. CAUTI incidence density was calculated as the number of CAUTI episodes per 1000 urinary catheter-days. CLABSI incidence density was calculated as the number of CLABSI episodes per 1000 central venous catheter-days. For CSF infections, incidence density was calculated separately per 1000 EVD-days and per 1000 lumbar drain-days when the corresponding exposure data were available. Device-days were calculated as the total number of calendar days during which the corresponding device was in place, summed across all periods of device exposure in patients who underwent device removal and subsequent reinsertion. Each infection episode was individually reviewed against the predefined device-association criteria before being assigned to a device-associated category, and only episodes fulfilling these criteria were included in the respective device-day-normalized rates.

Device utilization ratios were calculated as the number of device-days divided by the total number of ICU patient-days and expressed as a percentage. Separate utilization ratios were calculated for mechanical ventilation, urinary catheters, central venous catheters, EVDs, and LDs. The following formulas were applied:

Frequency (%) = number of infection episodes/total number of patients × 100

Incidence rate = number of infection episodes/total patient-days (hospital or ICU) × 1000

### 2.7. Microbiological Analysis

All microbiological analyses were performed at the Microbiology Laboratory of the University Clinical Hospital in Wroclaw. Multiplex polymerase chain reaction (PCR) assays were used, when clinically indicated, as an adjunct to conventional microbiological diagnostics for rapid pathogen identification. Molecular testing was performed using the BioFire FilmArray platform (BioFire Diagnostics, LLC, Salt Lake City, UT, USA; a bioMérieux company). Respiratory pathogens were assessed using the BioFire FilmArray Respiratory Panel and the BioFire FilmArray Pneumonia Panel, depending on the type of respiratory specimen and clinical indication. Bloodstream pathogens were identified from positive blood culture samples using the BioFire FilmArray Blood Culture Identification (BCID) Panel, whereas central nervous system pathogens in cerebrospinal fluid (CSF) were assessed using the BioFire FilmArray Meningitis/Encephalitis (ME) Panel.

Conventional microbiological cultures were performed for definitive pathogen identification and antimicrobial susceptibility testing. Susceptibility results were interpreted according to the European Committee on Antimicrobial Susceptibility Testing (EUCAST) breakpoints applicable at the time of testing and relevant European and national microbiological standards [43]. Molecular assays were used as complementary diagnostic methods and were not considered stand-alone criteria for the diagnosis of healthcare-associated infections.

Multidrug-resistant bacterial isolates were categorized according to the internationally accepted definitions proposed by Magiorakos et al. [44]. Alert pathogens were defined as multidrug-resistant (MDR) or extensively drug-resistant (XDR) Gram-negative bacteria, including Enterobacterales, *Pseudomonas aeruginosa*, and *Acinetobacter* spp., as well as methicillin-resistant *Staphylococcus aureus* (MRSA) and glycopeptide-resistant enterococci (GRE).

### 2.8. Outcome Measures

Functional neurological outcome at hospital discharge was assessed using the Glasgow Outcome Scale (GOS). Outcomes were dichotomized as unfavorable (GOS 1–3) and favorable (GOS 4–5), with higher scores indicating better functional status [45].

Final hospital disposition was categorized as discharge home, transfer to a rehabilitation facility, transfer to another hospital, transfer to a long-term care facility, or in-hospital death.

Other clinical outcomes included in-hospital mortality, time to live hospital discharge, ICU length of stay, total hospital length of stay, duration of mechanical ventilation, occurrence of DCI, and TCD-detected cerebral vasospasm.

Ethical review and approval were waived for this study because of its purely retrospective and non-interventional design and the exclusive use of fully anonymized clinical data routinely collected during hospitalization. The Bioethics Committee of Wroclaw Medical University confirmed in writing that separate ethical approval was not required and that the present analysis fell within the scope of its favorable opinion No. KB-620/2020, dated 12 October 2020.

### 2.9. Statistical Analysis

Continuous variables were summarized as medians with interquartile ranges (IQRs) and compared using the Mann–Whitney U test. Categorical variables were summarized as counts and percentages and were compared using the chi-square or Fisher’s exact test, as appropriate. Proportions are reported with Wilson 95% confidence intervals (CIs).

The burden of HAI was described at both the patient and episode levels. The proportion of patients developing at least one HAI, the total number of infection episodes, and the number of episodes per affected patient were reported. Incidence densities were expressed per 1000 patient-days or corresponding device-days, with exact Poisson 95% CIs.

#### 2.9.1. Analysis of Time to First HAI

Time to first HAI was analyzed using a cause-specific Cox proportional hazards model. For this analysis, the time origin was the date of admission to the neurocritical care unit. Patients were followed until the first HAI, hospital discharge without HAI, or in-hospital death. Discharge without HAI and death were treated as competing events. Cumulative incidence functions were estimated using the Aalen–Johansen method.

Age, sex, and WFNS grade were pre-specified as covariates based on clinical relevance and established associations with disease severity. Variables occurring after HAI onset were not included as baseline predictors. Length of stay and cumulative device-days were therefore excluded from baseline predictive models because they incorporated information occurring after or during the risk period for HAI. The Fisher grade was evaluated only in sensitivity analysis because of complete separation. Additional sensitivity analyses replaced the WFNS grade with the admission GCS, FOUR score, or APACHE II score. Results are reported as hazard ratios (HRs) with 95% CIs.

#### 2.9.2. Analysis of HAI as a Time-Dependent Exposure

To minimize immortal-time bias, HAI was modeled as a time-dependent exposure in analyses evaluating its association with subsequent clinical outcomes. Patients were considered unexposed until the date of the first HAI and exposed thereafter. In-hospital mortality was analyzed using Cox proportional hazards regression adjusted for age and WFNS grade. Hospital length of stay was analyzed as the time to live discharge using a cause-specific Cox model with death as a competing event. ICU length of stay and duration of mechanical ventilation were analyzed descriptively.

#### 2.9.3. Neurological Outcomes

Neurological outcome at hospital discharge was assessed using the full five-level GOS. An ordinal regression model was initially considered. However, because the proportional odds assumption was violated, a partial proportional odds model was used. The model accounted for the non-proportionality of the effect across outcome thresholds. In addition, dichotomized GOS analyses were performed as sensitivity analyses. The primary dichotomization defined an unfavorable outcome as GOS 1–3 and a favorable outcome as GOS 4–5. A further threshold-based analysis was performed to examine whether the association between HAI and outcome differed according to the severity of functional disability. A day-10 landmark analysis was performed as an additional sensitivity analysis to reduce potential bias arising from the different times at which HAI could occur. Only patients alive and hospitalized at the landmark time were included in this analysis, and HAI status by day 10 was used to define exposure. Hospital discharge destination was analyzed as an ordinal outcome in a sensitivity analysis.

#### 2.9.4. Additional Analyses

DCI was incorporated as a time-dependent covariate in a sensitivity analysis of mortality to account for the temporal relationship among DCI, HAI, and subsequent outcome. Device exposure was described for each infection type, and device duration was additionally examined using time-dependent covariates where appropriate.

Device exposure was summarized as the number of exposed patients, total device-days, and median exposure duration. The CAUTI incidence density was calculated per 1000 urinary catheter-days, and VAP, CLABSI, and CSF infection rates were normalized to ventilator-days, central venous catheter-days, and drain-days, respectively. Every episode was reviewed against the corresponding device-association criteria before classification, and device exposure was verified from the recorded device-exposure period.

The proportional hazards assumption was assessed using Schoenfeld residuals. Firth penalized estimation was applied in the presence of separation. Univariable analyses were exploratory and adjusted for multiple testing using the Benjamini–Hochberg method. Complete-case analysis was performed without imputation. All statistical tests were two-sided, with *p* < 0.05 considered statistically significant. Statistical analyses were performed in R version 4.5.2 using the survival, cmprsk, VGAM, brant, coxphf, and logistf packages. Statistical analyses and reporting were conducted in accordance with the STROBE (Strengthening the Reporting of Observational Studies in Epidemiology) recommendations for observational studies.

## 3. Results

### 3.1. Baseline Characteristics of the Study Cohort

A total of 106 patients with aneurysmal subarachnoid hemorrhage (aSAH) were included in the analysis. The median age was 54.5 years (IQR, 44.0–65.8), and 57 patients (53.8%) were women.

The cohort was characterized by substantial neurological severity at admission. WFNS grades IV–V were present in 57 patients (53.8%), while 96 patients (90.6%) had high Fisher grades (3–4). The median admission GCS was 13 (IQR, 5–15), and the median APACHE II score was 21 (IQR, 13–27). The median FOUR score was 12.5 (IQR, 7–16).

Endovascular treatment was performed in 73 patients (68.9%), whereas 33 patients (31.1%) underwent microsurgical clipping. Cerebrospinal fluid drainage was required in a substantial proportion of patients, including external ventricular drainage (EVD) and/or lumbar drainage (LD), according to clinical indications.

Mechanical ventilation was required in 91 patients (85.8%), and tracheostomy was performed in 29 patients (27.4%). The median duration of mechanical ventilation was 8 days (IQR, 2–16.8), while the median durations of urinary catheterization and central venous catheter exposure were 12.5 days (IQR, 7–19) and 10 days (IQR, 6–18), respectively.

The median ICU length of stay was 13.5 days (IQR, 8–20), and the median total hospital length of stay was 19 days (IQR, 14–43.2).

DCI occurred in 61 of 101 patients with available data (60.4%), with a median onset on day 6 after ictus (IQR, 4–8). TCD-detected vasospasm was identified in 62 of 105 patients (59.0%). Hydrocephalus occurred in 22 patients (20.8%), and seizures were documented in 21 patients (19.8%).

At hospital discharge, 40 patients (37.7%) had a favorable neurological outcome (GOS 4–5), whereas 66 patients (62.3%) had an unfavorable outcome (GOS 1–3). In-hospital mortality was observed in 31 patients (29.2%).

Detailed demographic, clinical, radiological, treatment, and outcome characteristics are presented in Table 1.

### 3.2. Infection Profile in the Study Cohort

#### 3.2.1. Time to First HAI

Among the 106 patients included in the study, 47 (44.3%; 95% CI, 35.2–53.8%) developed at least one HAI during hospitalization. Among these patients, the median time from ICU admission to the first HAI was 10 days (IQR, 8–12; range, 4–29 days). No initial HAI occurred during the first 3 days of hospitalization. A total of 11 patients (23.4%) developed their first HAI between days 4 and 7, 26 patients (55.3%) between days 8 and 14, and 10 patients (21.3%) after day 14. Thus, more than half of all first HAIs occurred during the second week of hospitalization.

The timing of infection varied according to infection type. VAP was diagnosed in 30 patients, with a median onset of 12 days (IQR, 10–17; range, 5–31 days). CAUTI occurred in 24 patients, with a median onset of 12 days (IQR, 9–19.5; range, 4–35 days). CLABSI occurred in 9 patients, with a median onset of 11 days (IQR, 10–21; range, 6–36 days), whereas CSF infection occurred in 13 patients, with a median onset of 12 days (IQR, 8–15; range, 4–28 days). All pneumonia episodes occurred in patients in whom invasive mechanical ventilation was in place within the 48 h preceding infection onset, including four episodes occurring after reintubation following a period without invasive ventilatory support. No episode of hospital-acquired pneumonia occurring independently of invasive mechanical ventilation was identified.

The distribution and timing of infectious complications are presented in Table 2.

#### 3.2.2. Frequency and Burden of Infection

HAIs occurred in 47 of 106 patients (44.3%), whereas 59 patients (55.7%) remained free of infectious complications during hospitalization. Among patients with HAI, 24 (51.1%) experienced a single infectious episode, while 23 (48.9%) developed two or more episodes.

Overall, 76 HAI episodes were recorded in the cohort. Across the entire cohort, the mean number of HAIs per patient was 0.72 (median, 0; IQR, 0–1). Among patients who developed at least one HAI, the median number of episodes was 1 (IQR, 1–2), with a mean of 1.62 episodes per patient.

The cohort contributed a total of 3507 hospital patient-days and 1650 ICU patient-days. The overall incidence density was 21.7 HAI episodes per 1000 hospital patient-days (95% CI, 17.1–27.1) and 46.1 episodes per 1000 ICU patient-days (95% CI, 36.3–57.7).

**Figure 2:**
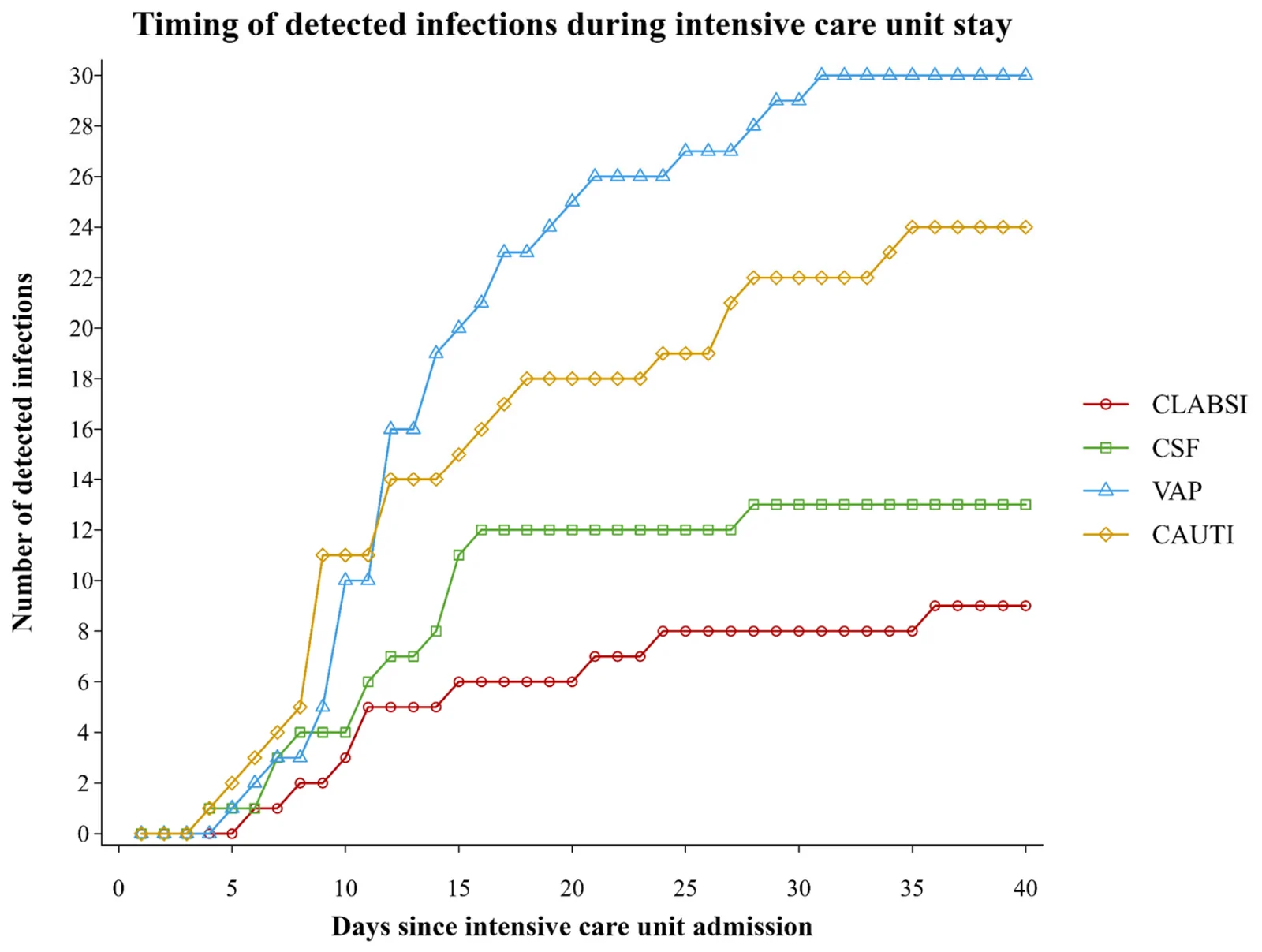
Cumulative timing of healthcare-associated infections by infection type during intensive care unit hospitalization. CLABSI—central line-associated bloodstream infection; CSF—cerebrospinal fluid infection; VAP—ventilator-associated pneumonia; CAUTI—catheter-associated urinary tract infection. Own elaboration using R statistical software, version 4.5.2.

Device-associated infection densities and corresponding device-day denominators are presented in Supplementary Table S1. Figure 2 illustrates the cumulative occurrence of HAI episodes according to infection type during the ICU stay.

VAP was the most frequent HAI, affecting 30 patients (28.3%) and accounting for 39.5% of all infectious episodes. CAUTI occurred in 24 patients (22.6%; 31.6% of all episodes), followed by CSF infection in 13 patients (12.3%; 17.1%) and CLABSI in 9 patients (8.5%; 11.8%).

The annual distribution of HAIs is presented in Table 3. The proportion of patients experiencing at least one HAI varied across the study period, ranging from 33.3% in 2024 to 61.5% in 2021. VAP remained one of the predominant infection types throughout the study period, whereas CLABSI was uncommon, and no CLABSI episodes were recorded in 2023 or 2024.

#### 3.2.3. Characteristics of Specific Infections

Device exposure was substantial during ICU treatment. Mechanical ventilation was used in 91 of 106 patients (85.8%), accounting for 1145 ventilator-days. All 106 patients underwent urinary catheterization and central venous catheterization, accounting for 1623 urinary catheter-days and 1371 central venous catheter-days, respectively; the corresponding median exposure durations were 12.5 and 10 days. Cerebrospinal fluid drainage was used in 53 patients (50.0%) for a total of 373 drain-days, including 214 EVD-days and 159 lumbar drain-days.

##### Ventilator-Associated Pneumonia

**Figure 3:**
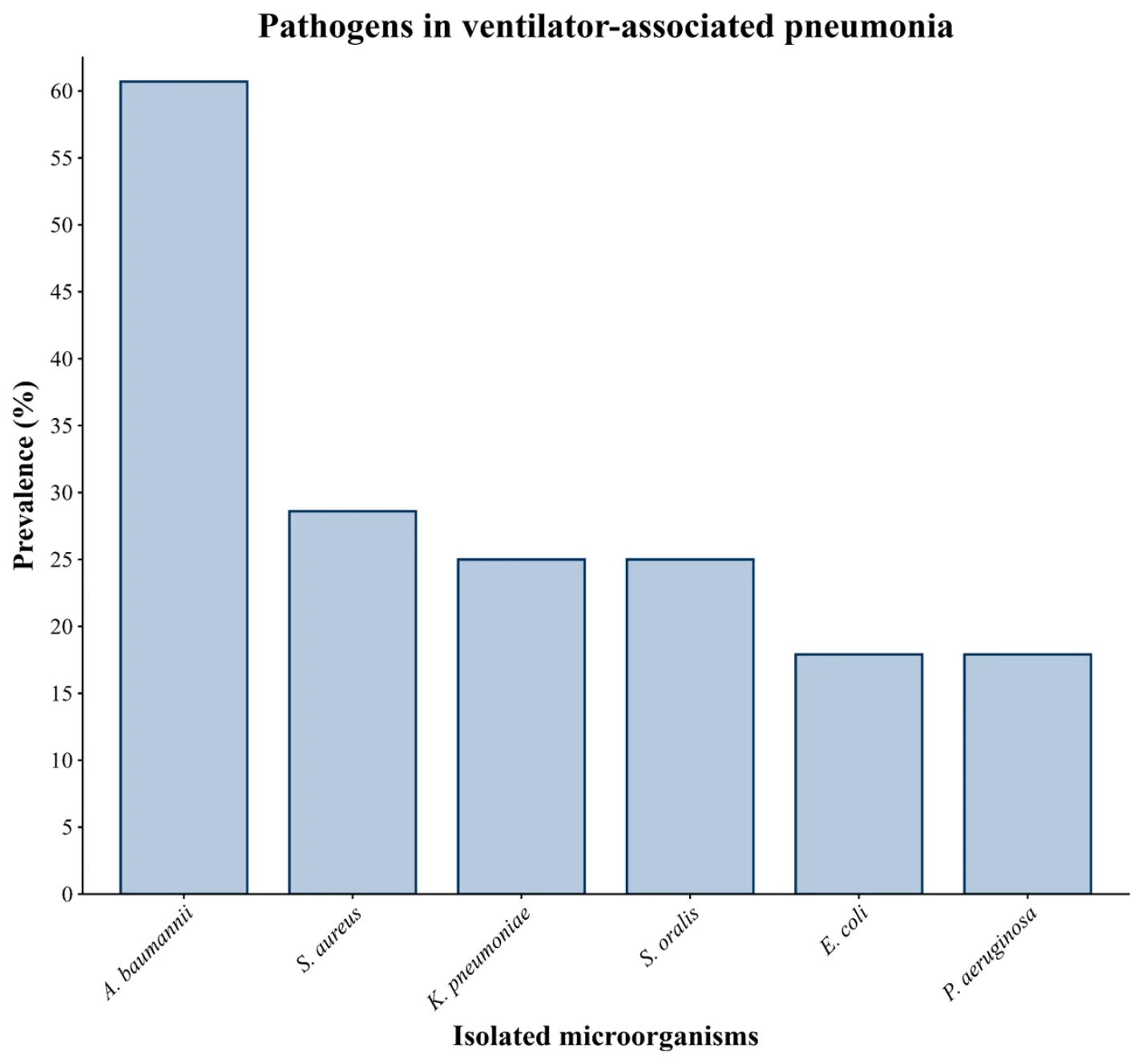
Six most frequently isolated pathogens in ventilator-associated pneumonia. Own elaboration using R statistical software, version 4.5.2.

VAP was diagnosed in 30 patients (28.3% of the cohort), accounting for 39.5% of all infectious episodes. The median time to diagnosis was 12 days [IQR, 7 days]. All patients with VAP required mechanical ventilation, and 20 (66.7%) underwent tracheostomy, performed at a median of 10.5 days [IQR, 9–20] after ICU admission. The median duration of mechanical ventilation was 20.5 days, compared with 4 days in patients without VAP. *Acinetobacter baumannii* was the most frequently isolated pathogen, identified in 17 patients (56.7%), followed by *Staphylococcus aureus* (8 patients, 26.7%), *Klebsiella pneumoniae* (7 patients, 23.3%), *Streptococcus oralis* (7 patients, 23.3%), and both *Escherichia coli* and *Pseudomonas aeruginosa* (5 patients each, 16.7%). The six most frequently isolated pathogens are presented in Figure 3, whereas the complete microbiological profile is provided in Supplementary Table S2.

Mechanical ventilation duration was significantly longer in patients with VAP than in those without VAP (median 20.5 days [IQR, 15–29.8] vs. 4 days [IQR, 2–10], *p* < 0.001). Based on 1145 ventilator-days, the VAP incidence density was 26.2 episodes per 1000 ventilator-days (95% CI, 17.7–37.4).

##### Catheter-Associated Urinary Tract Infection

CAUTI was diagnosed in 24 patients (22.6% of the cohort), accounting for 31.6% of all infectious episodes. The median time to diagnosis was 12 days (IQR, 10.5 days). All patients with CAUTI required mechanical ventilation, and 14 (58.3%) underwent tracheostomy. The median duration of mechanical ventilation was 19 days.

All 106 patients had an indwelling urinary catheter during ICU treatment, yielding 1623 urinary catheter-days (median, 12.5 days per patient). Urinary catheter duration was significantly longer in patients who developed CAUTI than in those without CAUTI (median 28 days [IQR, 17.8–34.5] vs. 10 days [IQR, 6–16], *p* < 0.001). As all CAUTI episodes occurred in catheterized patients and met the predefined criteria, the CAUTI incidence density was 14.8 episodes per 1000 urinary catheter-days (95% CI, 9.5–22.0).

**Figure 4:**
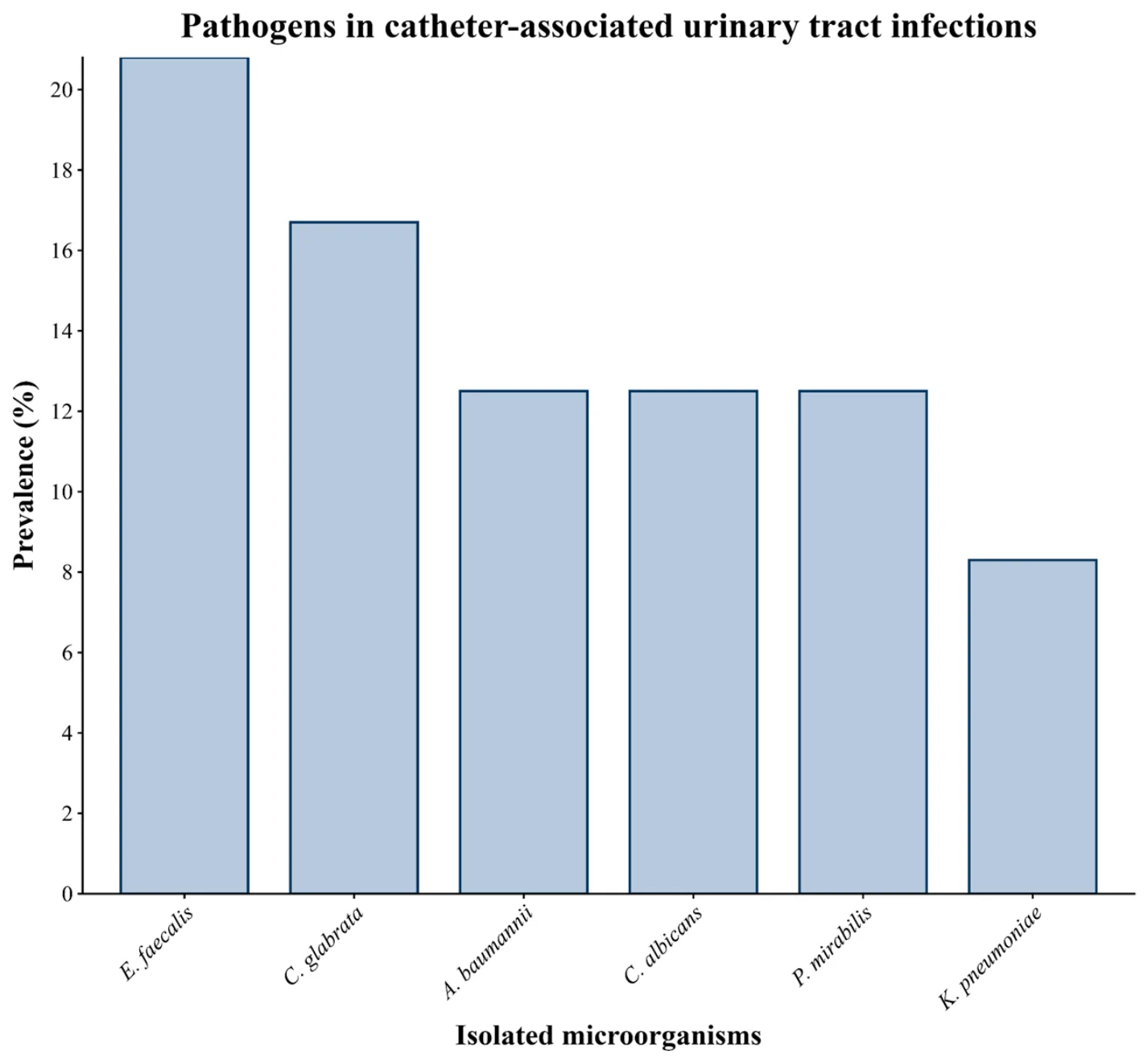
Six most frequently isolated pathogens in catheter-associated urinary tract infections. Own elaboration using R statistical software, version 4.5.2.

Urine cultures were positive in all patients with CAUTI. The most frequently identified pathogen was *Enterococcus faecalis* (five patients, 20.8%), followed by *Candida glabrata* (four patients, 16.7%), while *Acinetobacter baumannii*, *Candida albicans*, and *Proteus mirabilis* were identified in three patients each (12.5%). All *Candida* isolates fulfilled the predefined diagnostic criteria for symptomatic CAUTI. The six most frequently isolated pathogens are shown in Figure 4, whereas the complete microbiological profile is provided in Supplementary Table S2.

##### Cerebrospinal Fluid Infection

CSF infection was diagnosed in 13 patients (12.3% of the cohort) and accounted for 13 infectious episodes (17.1%). The median time to diagnosis was 12 days (IQR, 7 days). All patients required mechanical ventilation, and 6 (46.2%) underwent tracheostomy. The median duration of mechanical ventilation was 19 days. CSF infection occurred in 11 patients exposed to cerebrospinal fluid drainage and in 2 patients without recorded drainage exposure. Among drainage-associated cases, seven occurred in patients with EVD and four in patients with lumbar drainage.

Among patients exposed to cerebrospinal fluid drainage, 27 were exposed to EVD for a total of 214 EVD-days and 26 to lumbar drainage for 159 drain-days. Seven CSF infections occurred in patients with EVD exposure, corresponding to 32.7 infections per 1000 EVD-days (95% CI, 13.2–67.4). Four CSF infections occurred during lumbar drain exposure, corresponding to 25.2 infections per 1000 lumbar drain-days (95% CI, 6.9–64.4). Device-day-normalized rates describe the occurrence of CSF infection during periods of documented EVD or lumbar drain exposure and should not be interpreted as formal EVD-associated or lumbar drain-associated infection rates unless all episodes fulfill the corresponding device-association criteria.

**Figure 5:**
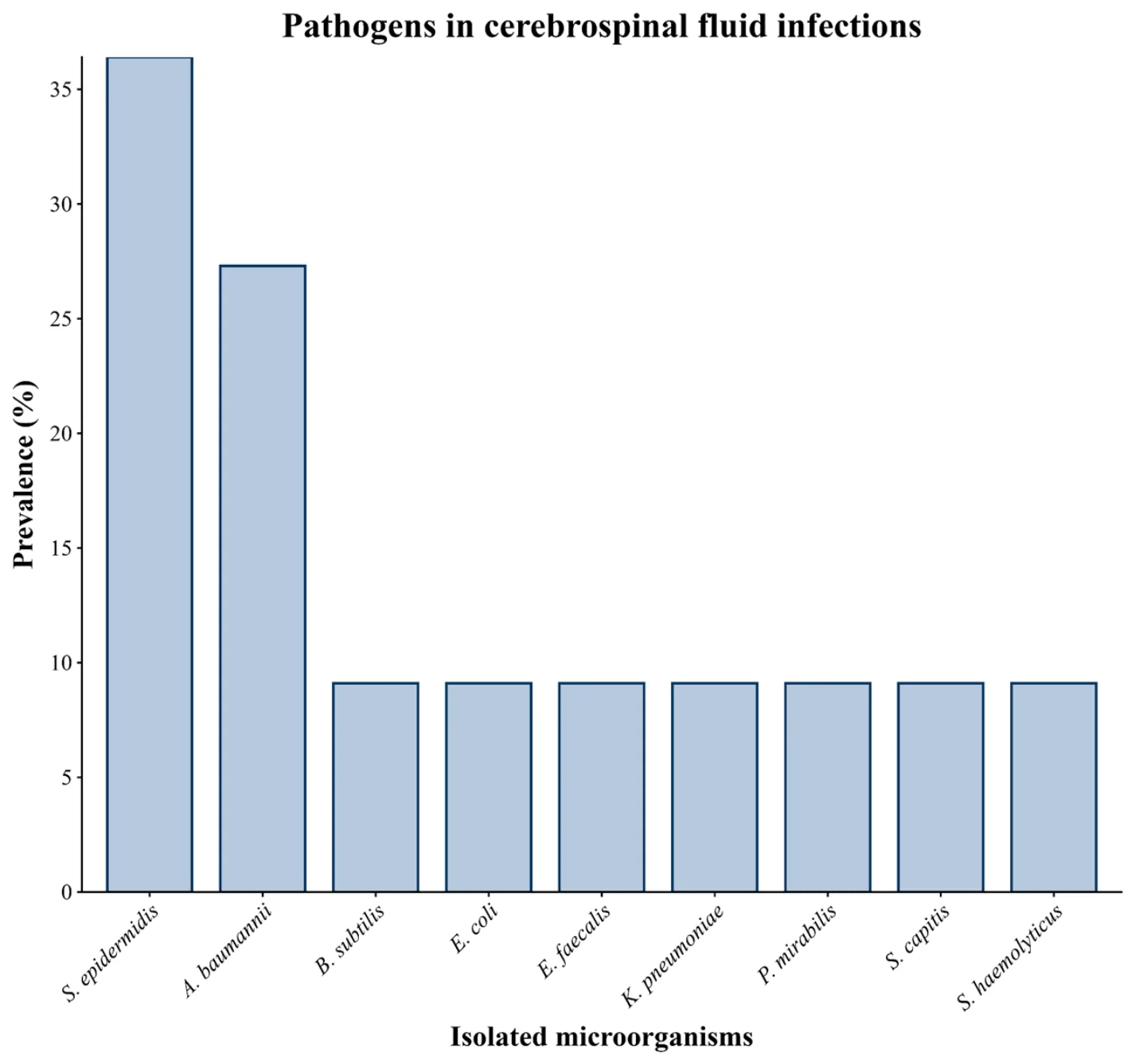
Distribution of pathogens isolated from cerebrospinal fluid infections. Own elaboration using R statistical software, version 4.5.2.

CSF cultures yielded a causative pathogen in 11 of 13 patients (84.6%), whereas microbiological results were unavailable in two patients. *Staphylococcus epidermidis* was the most frequently isolated microorganism, identified in four patients (30.8%), followed by *Acinetobacter baumannii* in three patients (23.1%). Other pathogens were isolated in single patients. The complete microbiological profile is presented in Figure 5. Detailed microbiological profiles of all HAIs are provided in Supplementary Table S2.

##### Central Line-Associated Bloodstream Infection

CLABSI occurred in nine patients (8.5% of the cohort), accounting for 11.8% of all infectious episodes. The median time to diagnosis was 11 days (IQR, 11 days). All patients required mechanical ventilation, and six (66.7%) subsequently underwent tracheostomy. The median duration of mechanical ventilation was 28 days.

All 106 patients had a central venous catheter during ICU treatment, yielding 1371 central venous catheter-days (median, 10 days per patient). Central venous catheter duration was significantly longer in patients who developed CLABSI than in those without CLABSI (median 20 days [IQR, 16–25] vs. 10 days [IQR, 5–17], *p* = 0.0025). The CLABSI incidence density was 6.6 episodes per 1000 central venous catheter-days (95% CI, 3.0–12.5). All CLABSI episodes occurred in patients in whom a central venous catheter was in place within the 48 h preceding infection onset. In two patients, infection developed after reinsertion of a central venous catheter following a catheter-free interval. Each episode was individually adjudicated against the predefined device-association criteria before classification.

**Figure 6:**
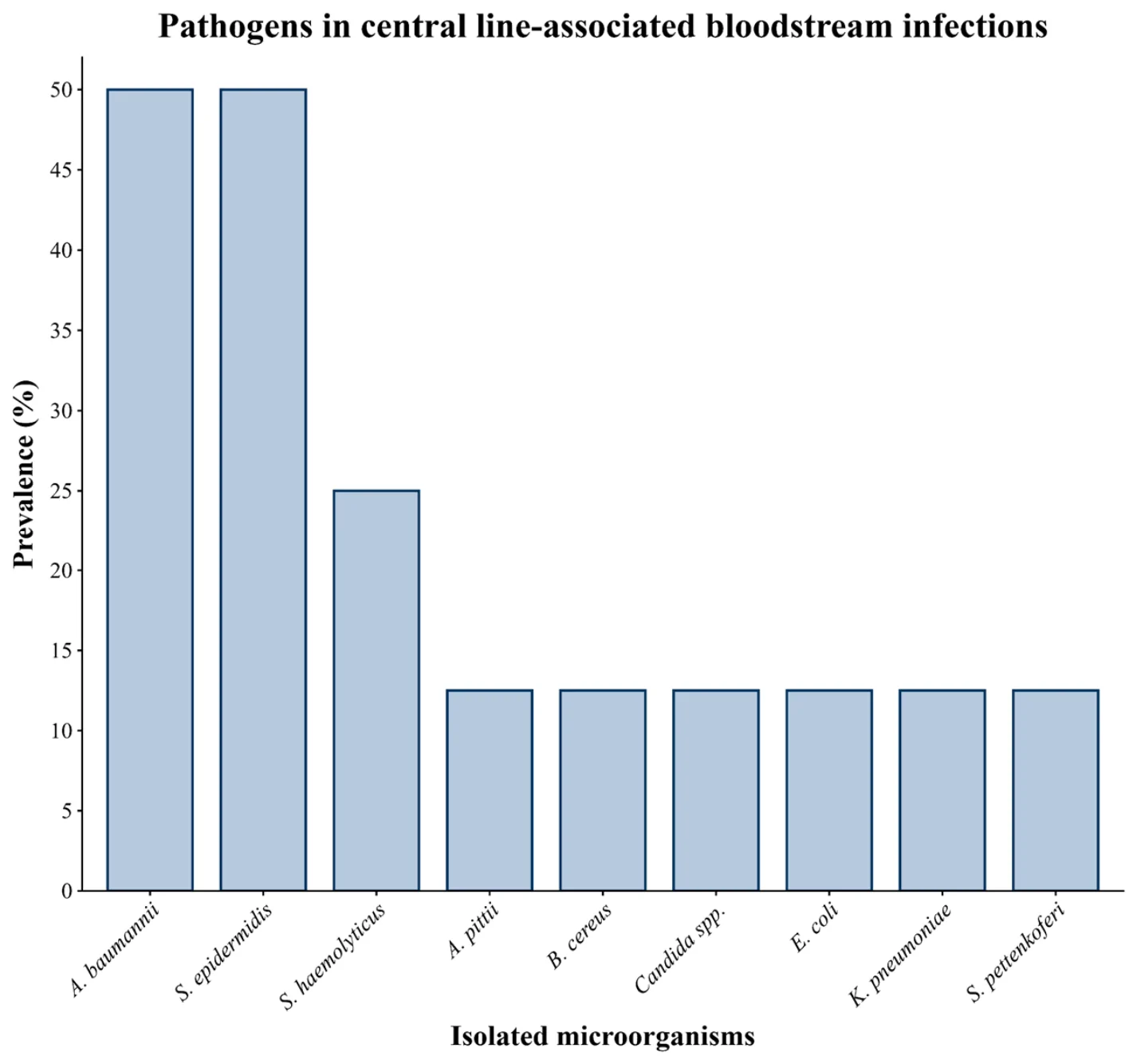
Distribution of pathogens isolated from central line-associated bloodstream infections. Own elaboration using R statistical software, version 4.5.2. The profile comprises the eight isolates identified to species level; a ninth isolate, documented only as an unidentified Gram-negative bacillus, is listed in Supplementary Table S2. Percentages are calculated from all nine CLABSI episodes.

Blood cultures were positive in all nine patients with CLABSI. Species-level identification was available in eight episodes (88.9%). In the remaining episode, the isolate was documented only as a Gram-negative bacillus, without further identification. It was therefore not included in the species-level profile shown in Figure 6 but is listed in Supplementary Table S2. *Acinetobacter baumannii* and *Staphylococcus epidermidis* were the most frequently identified pathogens, each identified in four patients (44.4%), followed by *Staphylococcus haemolyticus* (two patients, 22.2%). The microbiological profile of CLABSIs is presented in Figure 6.

Detailed microbiological profiles of all HAIs are provided in Supplementary Table S2.

#### 3.2.4. Co-Occurrence of Multiple Infection Types

Among the 47 patients who developed at least one HAI, 24 patients (51.1% of infected patients; 22.6% of the entire cohort) experienced a single type of infection, whereas 23 patients (48.9% of infected patients; 21.7% of the entire cohort) developed more than one type of infection during hospitalization. Overall, 76 infectious episodes were recorded.

VAP occurred in 30 patients, CAUTI in 24, CSF infection in 13, and CLABSI in 9. Because individual patients could develop more than one infection type, these categories were not mutually exclusive, and the corresponding percentages should not be summed.

**Figure 7:**
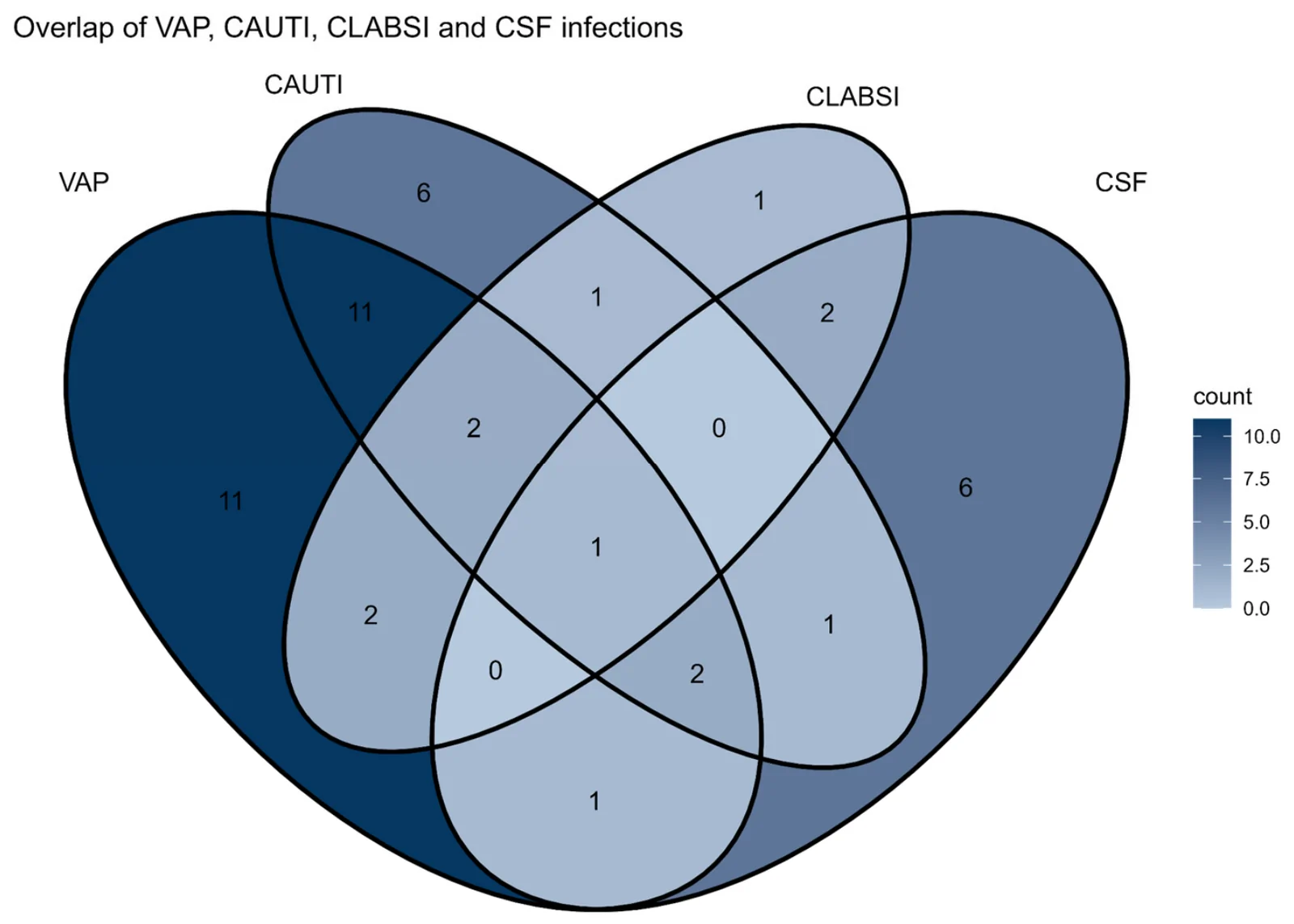
Patient-level overlap of VAP, CAUTI, CLABSI, and CSF infections in patients with aSAH. Own elaboration using R statistical software, version 4.5.2.

Among patients with multiple infection types, 18 developed two different infection types, 4 developed three types, and 1 patient experienced all four types. The most frequent combination was VAP with CAUTI, observed in 11 patients. VAP with CLABSI and CLABSI with CSF infection each occurred in two patients, whereas VAP with CSF infection, CAUTI with CLABSI, and CAUTI with CSF infection were each observed in one patient. Two patients developed VAP, CAUTI, and CLABSI, and another two developed VAP, CAUTI, and CSF infection. One patient experienced all four infection types—VAP, CAUTI, CLABSI, and CSF infection—during hospitalization. The patient-level overlap between infection types is illustrated in Figure 7.

To further explore whether infection burden was associated with the subsequent clinical course, patients with a single HAI type were compared with those who developed two or more HAI types. The results are presented in Table 4.

Patients who developed multiple HAI types experienced a more prolonged and intervention-intensive ICU course, characterized by longer mechanical ventilation, longer urinary catheter exposure, longer ICU and hospital stays, and a greater frequency of tracheostomy. In contrast, the number of HAI types was not significantly associated with DCI, neurological outcome at discharge, or in-hospital mortality. Given the relatively small subgroup sizes, these comparisons should be considered exploratory and the absence of statistically significant differences in clinical outcomes should not be interpreted as evidence of equivalence.

#### 3.2.5. Antimicrobial Resistance Among Patients with HAI 

Antimicrobial resistance was common among isolates from patients with HAI. *Acinetobacter baumannii*, the most frequently isolated bacterial pathogen in the cohort, was also the organism most strongly associated with advanced resistance phenotypes, accounting for all XDR isolates and for most MDR phenotypes. Among Gram-positive organisms, resistance was predominantly observed in staphylococci, with macrolide–lincosamide–streptogramin B (MLSB) resistance and methicillin-resistant staphylococci representing the most frequent phenotypes. MRSA was also identified among *S. aureus* isolates. Detailed organism-specific resistance patterns are presented in Table 5.

### 3.3. Comparison of Patients with and Without Infection

Patients were stratified according to the occurrence of HAI during hospitalization. The non-infection group comprised 59 patients, whereas 47 patients developed at least one HAI.

No significant between-group differences were observed regarding age, sex, BMI, or chronic comorbidities (Table 6).

In contrast, patients who developed HAI presented with significantly greater neurological and clinical severity at admission. They had lower GCS scores (median 8 vs. 14, *p* = 0.023), higher WFNS grades (median 5 vs. 2, *p* < 0.001), higher Fisher scores (*p* < 0.001), and higher APACHE II scores during the ICU stay (median 24 vs. 14, *p* < 0.001). Differences in intensive care treatment, hospitalization course, and clinical outcomes according to infection status are summarized in Table 7.

Treatment-related variables and the subsequent clinical course differed significantly between groups. Patients who developed infection more frequently required mechanical ventilation (100% vs. 74.6%, *p* < 0.001), underwent tracheostomy more often (51.1% vs. 8.5%, *p* < 0.001), and had longer durations of mechanical ventilation (median 18 vs. 3 days, *p* < 0.001). They also more frequently required vasoactive support (40/59, 67.8% vs. 44/47, 93.6%; *p* = 0.001).

Patients with HAI had significantly longer ICU stays (median 21 vs. 8 days, *p* < 0.001) and total hospital stays (median 44 vs. 18 days, *p* < 0.001). They also had worse neurological outcomes at discharge, with lower GOS scores (median 2 vs. 4, *p* < 0.001) and a higher proportion of unfavorable outcomes (87.2% vs. 42.4%, *p* < 0.001). Although in-hospital mortality did not differ significantly between groups (*p* = 0.451), discharge destinations differed; patients with infection were more frequently transferred to rehabilitation facilities.

### 3.4. Exploratory Associations of DCI and TCD-Detected Vasospasm with HAI

Delayed cerebral ischemia was diagnosed in 61 of 101 assessable patients (60.4%); DCI status could not be determined in 5 patients whose neurocritical care course ended within the first days after admission, before the period in which DCI typically develops, and who all died during hospitalization. HAI occurred in 37 of 61 patients with DCI (60.7%) compared with 9 of 40 patients without DCI (22.5%). Because DCI is a time-dependent complication developing during the clinical course rather than a baseline characteristic, this comparison was considered exploratory.

Among the patients who experienced both DCI and HAI (*n* = 37), DCI preceded the first HAI episode in 27 cases (73.0%), whereas HAI preceded DCI in 10 cases (27.0%); no DCI events occurred on the same day. The median onset of DCI in this subgroup was day 7 (IQR, 5–9), while the first HAI occurred at a median of day 10 (IQR, 8–12). When individual infection types were considered, 51 of 62 infectious events (82.3%) developed after DCI onset, whereas 11 (17.7%) events occurred before DCI. The temporal distribution of infectious events relative to DCI onset is shown in Table 8.

TCD-detected cerebral vasospasm was also more frequent among patients who developed HAI than among those without HAI (36/47 [76.6%] vs. 26/58 [44.8%], *p* = 0.002). Because the temporal sequence between TCD-detected vasospasm and HAI was not evaluated, this finding should be interpreted as an exploratory association rather than evidence of a temporal or causal relationship. Similarly, the observed temporal relationship between DCI and HAI should not be interpreted as evidence that DCI independently increases infection risk, because DCI, vasospasm, and HAI may all be associated with greater neurological severity, prolonged intensive care, and invasive treatment exposure.

### 3.5. Risk Factors for Infection Occurrence

Time to first HAI was analyzed using a cause-specific Cox proportional hazards model, with death and discharge without infection treated as competing events. In the primary adjusted model, high WFNS grade (IV–V) was strongly associated with a higher hazard of first HAI (HR = 4.168, 95% CI 2.072–8.384; *p* < 0.001). Neither age nor sex was significantly associated with the risk of developing HAI. Sensitivity analyses using alternative measures of neurological and clinical severity yielded consistent findings: lower GCS (HR = 0.921, 95% CI 0.865–0.981; *p* = 0.011) and FOUR scores (HR = 0.889, 95% CI 0.832–0.951; *p* < 0.001), as well as higher APACHE II scores (HR = 1.072, 95% CI 1.034–1.112; *p* < 0.001). Similarly, a higher Fisher score was associated with an increased risk of first HAI (HR, 2.886; 95% CI, 1.237–6.735; *p* = 0.014). Taken together, these findings consistently indicate that greater neurological and clinical severity at admission was associated with a higher subsequent hazard of developing HAI. The complete results of the primary and sensitivity analyses are presented in Table 9.

### 3.6. Impact of Infection on Hospital Course and Clinical Outcome

The association between HAI and subsequent clinical outcomes was further evaluated using time-to-event and ordinal regression models that accounted for the time-dependent nature of infection. In the time-dependent Cox model adjusted for age and WFNS grade, HAI was not significantly associated with in-hospital mortality (HR = 1.178, 95% CI 0.478–2.902; *p* = 0.721). Hospital length of stay was analyzed as time to live discharge, with death treated as a competing event. Time-dependent HAI was significantly associated with a lower hazard of live discharge (HR = 0.295, 95% CI 0.164–0.529; *p* < 0.001), consistent with prolonged hospitalization among patients who developed infection. ICU length of stay and duration of mechanical ventilation were analyzed descriptively. The association between HAI and neurological outcome at discharge was examined using a partial proportional odds model because the proportional odds assumption was violated. The effect of HAI therefore varied across GOS thresholds. In a sensitivity analysis using dichotomized GOS, HAI was associated with an unfavorable outcome (OR = 6.373, 95% CI 2.110–21.904; *p* = 0.002), and the day-10 landmark analysis yielded a similar association (OR = 4.658, 95% CI 1.242–22.939; *p* = 0.033). Taken together, the time-dependent analyses indicate that HAI was associated with prolonged hospitalization and worse neurological outcome at discharge, but not with a statistically significant increase in in-hospital mortality after adjustment for age and baseline neurological severity. Complete regression results are presented in Table 10 and Table 11.

## 4. Discussion

### 4.1. Principal Findings and Their Clinical Implications

Our findings indicate that HAIs are a frequent complication among patients with aSAH requiring intensive care. Nearly half of the study cohort developed at least one HAI, and multiple infectious episodes were common. Most infections occurred after the first week of hospitalization, suggesting that their development was primarily associated with prolonged intensive care, exposure to invasive procedures, and extended use of medical devices, rather than with the initial hemorrhagic event itself.

VAP was the predominant infectious complication, followed by CAUTI, CSF infection, and CLABSI. The predominance of VAP is clinically relevant and, together with the universal requirement for mechanical ventilation among affected patients, emphasizes the importance of respiratory complications in this patient population.

Patients who developed HAIs had greater neurological and systemic disease severity at baseline. In the primary cause-specific Cox model, high WFNS grade was strongly associated with a higher hazard of first HAI. Importantly, sensitivity analyses using alternative measures of neurological and clinical severity yielded consistent findings: lower GCS and FOUR scores and higher APACHE II and Fisher scores were also associated with a higher hazard of first HAI. Taken together, these findings provide consistent evidence that greater initial neurological and disease severity was associated with subsequent HAI development. Importantly, the severity scales evaluated in this study are strongly interrelated and should be interpreted primarily as complementary markers of the severity of the initial neurological and systemic insult rather than as independent causal determinants of infection.

An additional exploratory finding was the relationship between HAI and secondary neurological complications. HAI occurred more frequently among patients who developed DCI than among those without DCI. Among patients who experienced both complications, DCI preceded the first HAI in nearly three-quarters of cases, with DCI occurring earlier during hospitalization than the first infectious complication. TCD-detected cerebral vasospasm was also more frequent among patients who developed HAI. These observations should not, however, be interpreted as evidence that DCI or vasospasm independently increases the risk of infection. DCI, vasospasm, and HAI may instead represent interrelated features of a more severe and prolonged neurocritical care course, characterized by greater neurological injury, longer exposure to invasive devices, and increased need for organ support. Moreover, because HAI preceded DCI in a proportion of patients and the temporal sequence between vasospasm and HAI was not evaluated, the relationship between these complications is likely complex and potentially bidirectional.

Patients who developed HAIs had markedly longer durations of mechanical ventilation, urinary and vascular catheterization, and ICU stays in descriptive comparisons. In time-dependent analyses, HAI was associated with a lower hazard of live hospital discharge, whereas no statistically significant association with in-hospital mortality was observed. The association with neurological outcome at discharge was threshold-dependent in the partial proportional odds model. The relationship between HAI and unfavorable clinical outcomes is likely bidirectional. Greater neurological severity increases exposure to mechanical ventilation, invasive devices, and prolonged intensive care, whereas infection may further complicate the clinical course and delay recovery. The present retrospective analysis cannot determine whether HAI independently caused poorer outcomes or primarily identified patients with more severe and prolonged critical illness. The absence of a statistically significant association between HAI and in-hospital mortality should be interpreted cautiously, as the relatively small number of deaths and the wide confidence interval limit the precision of this estimate and do not exclude a clinically relevant association. Because HAI was modeled as a time-dependent exposure, the mortality analysis was designed to reduce immortal-time bias related to the delayed occurrence of infection. Nevertheless, residual time-dependent confounding cannot be excluded because of the retrospective observational design.

The magnitude of this bias can be quantified within the present cohort. When HAI was analyzed as a fixed exposure present from admission, as in conventional analyses, infection appeared to be associated with a lower hazard of in-hospital death (HR 0.356, 95% CI 0.158–0.806; *p* = 0.013)—an apparently protective effect that is biologically implausible and instead reflects the requirement to survive long enough for an infection to be diagnosed. Nine patients died within the first 10 days of hospitalization, corresponding to the median time to first infection, and none of them had developed an HAI. Modeling HAI as a time-dependent exposure removed this artifact entirely (HR 1.178, 95% CI 0.478–2.902; *p* = 0.721). This comparison illustrates why associations between HAI and mortality after aSAH derived from fixed-exposure analyses should be interpreted with caution and supports the use of time-dependent methods in future studies of infectious complications in neurocritical care. Overall, these findings indicate that HAIs are associated with greater morbidity and healthcare resource utilization following aSAH.

### 4.2. Comparison with Previous Studies

The proportion of HAIs identified in our cohort (44.3%) is in line with previous reports in patients with aSAH. Kashiwazaki et al. reported infectious complications in 42.3% of patients, whereas Kilgore et al. described an incidence of approximately 45% [11,15]. Although the overall incidence was comparable, the proportion of patients experiencing multiple infectious episodes differed between studies, most likely reflecting differences in study populations, infection definitions, and local intensive care practices.

The median interval to the first infection was 10 days, with most infections occurring between days 8 and 14 of admission. Comparable temporal patterns have been documented in previous investigations, supporting the interpretation that infectious complications are primarily related to prolonged intensive care exposure rather than the acute phase of aneurysmal hemorrhage itself [15,46]. This temporal pattern is clinically relevant because the period of increasing infection risk overlaps with the time during which patients with severe aSAH often require prolonged mechanical ventilation, enteral nutrition, urinary and central venous catheterization, and, in selected cases, CSF drainage.

VAP was the most common HAI in our cohort, affecting nearly one-third of patients. This observation aligns with earlier reports, although incidence rates vary between studies [11,46,47]. Such variation may result from differences in the definition of VAP, duration of mechanical ventilation, severity of neurological injury, tracheostomy practices, surveillance intensity, and local infection-prevention strategies. The microbiological profile of VAP in our cohort was notable for the predominance of *Acinetobacter baumannii*, whereas *Staphylococcus aureus*, *Haemophilus influenzae*, and *Streptococcus pneumoniae* have been reported more commonly elsewhere [46]. These discrepancies most likely reflect local microbiological epidemiology, antimicrobial resistance profiles, and infection prevention practices rather than differences in the underlying pathophysiology. The predominant pathogens causing HAIs vary geographically, reflecting differences in local epidemiology, antimicrobial exposure, and infection-control practices.

In ventilator-associated pneumonia (VAP), *Pseudomonas aeruginosa*, *Staphylococcus aureus*, and *Klebsiella pneumoniae* are among the most frequently isolated pathogens in European ICUs, whereas *Acinetobacter baumannii* has been reported as particularly prevalent in Polish ICUs [48,49,50]. Similarly, *Staphylococcus aureus*, *Pseudomonas aeruginosa*, and *Klebsiella pneumoniae* are commonly identified as leading VAP pathogens in American ICUs [51].

The high prevalence of *Acinetobacter baumannii* was therefore consistent with previously reported Polish epidemiological patterns and highlights the importance of considering local microbiological profiles when selecting empirical antimicrobial therapy. The microbiological profile of CLABSIs also varies geographically. In European ICUs, coagulase-negative staphylococci are among the most frequently isolated microorganisms in ICU-acquired CLABSIs, followed by *Enterococcus* spp., whereas the contribution of Gram-negative pathogens, particularly *Klebsiella* spp. and *Acinetobacter* spp., varies considerably between countries [48].

Polish studies have reported a relatively high contribution of *Enterococcus* spp., *Acinetobacter* spp., and *Klebsiella* spp. to ICU-acquired CLABSIs, with *Klebsiella pneumoniae* being particularly prominent among Gram-negative isolates [52]. In our general ICU, between 2020 and 2024, *Staphylococcus epidermidis* was the most frequently identified etiological agent of CLABSI, accounting for 33.3% of cases, while alert pathogens represented 34.26% of all CLABSI-associated microorganisms [53]. In contrast, surveillance data from American ICUs indicate that coagulase-negative staphylococci and *Enterococcus* spp. remain important causes of CLABSI, with *Candida* spp. also frequently identified [54].

CAUTI was the second most frequent HAI in the present study. The observed frequency is consistent with previous reports describing urinary tract infections as an important complication among critically ill patients with aSAH [47]. In our cohort, all patients were exposed to urinary catheterization, and the duration of catheterization was significantly longer among patients who developed CAUTI. This finding reinforces the importance of limiting urinary catheterization to clinically necessary indications and removing the device as early as clinically feasible.

Bloodstream and CSF infections, similarly to previous studies, occurred less frequently but remained clinically relevant because they were observed predominantly in patients requiring prolonged critical care and multiple invasive devices [15,47].

The association between HAIs and longer ventilatory support, prolonged ICU care, extended hospitalization, and poorer neurological outcome observed within the analyzed cohort is in line with prior evidence suggesting that infectious complications substantially increase morbidity following aSAH [47,55]. However, the present time-dependent analyses additionally highlight the importance of distinguishing the consequences of infection from the characteristics of patients who survive long enough and remain critically ill long enough to develop infection.

### 4.3. Mechanisms Underlying the Increased Risk of Infection Following aSAH

#### 4.3.1. Neuroinflammation and Systemic Immune Response

Following aneurysm rupture, blood degradation products and damage-associated molecular patterns activate microglia, astrocytes, endothelial cells, and infiltrating macrophages, promoting the release of pro-inflammatory cytokines, including IL-1β, IL-6, and TNF-α. This response may contribute to blood–brain barrier dysfunction, cerebral edema, microvascular impairment, SIRS, and secondary brain injury [56,57,58]. Systemic infection may further amplify this pre-existing inflammatory state through peripheral cytokine release, endothelial activation, and altered leukocyte trafficking. Persistent glial activation after acquired brain injury has also been associated with chronic neuronal network dysfunction and post-injury epileptogenesis [59,60]. However, these mechanisms were not directly assessed in the present study and should be considered a biological framework for interpreting the observed associations.

#### 4.3.2. Central Nervous System Injury-Induced Immunodepression

The initial hyperinflammatory response is followed by compensatory stimulation of the hypothalamic–pituitary–adrenal axis together with the autonomic nervous system, resulting in CNS injury-induced immunodepression (CIDS). This syndrome is characterized by lymphocyte apoptosis, impaired monocyte antigen presentation, and dysregulation affecting both innate and adaptive immunity, substantially increasing susceptibility to HAIs, particularly VAP and CAUTI [61,62,63].

#### 4.3.3. Intensive Care-Related Factors

Neurological impairment further increases infection risk by necessitating prolonged mechanical ventilation, urinary catheterization, central venous access, and external ventricular drainage. At the same time, differentiating sterile neuroinflammation from true bacterial infection remains challenging because fever, leukocytosis, elevated CRP, and even PCT may reflect brain injury rather than infection. Consequently, diagnosis relies on the combined assessment of clinical, microbiological, laboratory, and imaging data. These elements, together with prolonged ICU exposure, explain the high incidence of device-associated infections observed after aSAH [64,65,66,67].

### 4.4. Clinical Implications

The present findings have several implications for clinical practice. First, systematic surveillance for HAIs should be maintained throughout the ICU stay, with particular attention to patients with severe neurological impairment and prolonged exposure to invasive devices. The observation that high WFNS grade was strongly associated with the subsequent development of HAI suggests that neurological severity may help identify patients requiring particularly intensive infection surveillance.

Second, infection-prevention strategies should focus on minimizing modifiable exposure to invasive devices. This includes daily assessment of the continued need for mechanical ventilation, urinary and central venous catheters, and CSF drainage, together with consistent implementation of evidence-based device-specific prevention bundles.

Third, the high prevalence of antimicrobial resistance observed in the present cohort has important implications for empirical treatment. The predominance of XDR *A. baumannii*, together with the presence of MRSA, ESBL-producing Enterobacterales, carbapenemase-associated phenotypes, and other resistance mechanisms, highlights the importance of local microbiological surveillance and institution-specific antimicrobial susceptibility data when selecting empirical therapy.

Finally, clinicians should recognize that infection may represent both a complication and a marker of a more severe neurocritical care course. Infection in a high-risk patient should not be regarded as an inevitable consequence of disease severity; rather, severity should prompt closer surveillance, early recognition of clinical deterioration, and rigorous application of preventive measures. Conversely, the occurrence of HAI should not automatically be interpreted as the cause of subsequent poor neurological outcome because of the substantial potential for confounding by baseline severity and time-dependent treatment exposure.

### 4.5. Study Strengths and Limitations

This study has several important advantages, including the analysis of a homogeneous group of patients with aSAH treated according to a standardized institutional protocol in a dedicated neurocritical care unit. Comprehensive clinical and microbiological data enabled detailed characterization of HAIs with respect to their incidence, timing, microbiological etiology, and clinical consequences. Furthermore, multivariable regression analyses allowed adjustment for major indicators of disease severity when evaluating both risk factors for infection and their association with clinical outcomes.

However, the study also has several limitations. This study was retrospective and conducted in a single neurocritical care unit with a relatively limited sample size. The observed incidence, timing, and microbiological profile of HAIs may therefore reflect local patient selection, ICU organization, infection-prevention practices, and antimicrobial ecology. These factors limit the external validity of the findings and warrant confirmation in larger multicenter cohorts. In addition, the exclusion of patients admitted exclusively for brain-death determination, those undergoing delayed aneurysm treatment, and patients with rerupture or intraoperative aneurysm rupture may have selected a more homogeneous and potentially less complicated population, thereby further limiting the generalizability of the findings. Second, the moderate sample size reduced the statistical power, especially for analyses of less frequent infectious complications and predefined subgroups. Third, the study period encompassed the COVID-19 pandemic, during which the number of admissions for aSAH declined substantially compared with the pre-pandemic and post-pandemic years. Changes in healthcare organization, referral patterns, and ICU capacity during this period may have influenced patient selection, diagnostic pathways, and infection-control practices.

Although HAI was modeled as a time-dependent exposure in the analyses of in-hospital mortality and live hospital discharge to reduce immortal-time bias, residual time-dependent confounding cannot be excluded because of the retrospective observational design. ICU length of stay and duration of mechanical ventilation were analyzed descriptively and should not be interpreted as independent causal effects of infection. Functional outcome was assessed at hospital discharge rather than at a fixed follow-up time; therefore, the timing of GOS assessment differed between patients and may have been influenced by the occurrence of HAI and the duration of hospitalization. This informative timing limits the causal interpretation of the association between HAI and discharge GOS.

Data on antimicrobial exposure preceding infection, corticosteroid therapy, nutritional status, enteral feeding, and depth and duration of sedation were not available for systematic analysis, and residual confounding related to these factors cannot be excluded. Furthermore, with 47 first-infection events, the multivariable models were deliberately parsimonious; adding further covariates would have risked overfitting and unstable estimates. These variables therefore represent important targets for prospective evaluation rather than omissions that could have been addressed within the present dataset.

In one of the nine CLABSI episodes, blood cultures were positive, but the isolate was documented only as Gram-negative bacilli, without genus- or species-level identification. Although the episode fulfilled the predefined BSI and central line-association criteria, the isolate could not be included in the species-level microbiological profile, which therefore reflects the eight episodes with complete identification. The isolate is nevertheless listed in Supplementary Table S2 as an unidentified Gram-negative bacillus.

## 5. Conclusions

Healthcare-associated infections are frequent complications among patients with aSAH requiring intensive care and are associated with a more prolonged and resource-intensive clinical course. In time-dependent analyses, HAI was associated with a lower hazard of live hospital discharge, whereas no statistically significant association with in-hospital mortality was identified. Longer ICU stays and durations of mechanical ventilation were observed among patients with HAI in descriptive comparisons, while the association between HAI and neurological outcome at hospital discharge was threshold-dependent and should be interpreted cautiously. Greater initial neurological and systemic disease severity was consistently associated with a higher risk of developing HAI, with a high WFNS grade demonstrating the strongest association in the primary time-to-event analysis.

These findings highlight the importance of systematic HAI surveillance, timely microbiological diagnosis, and consistent implementation of evidence-based infection-prevention strategies throughout neurocritical care, particularly in patients with greater neurological severity and those requiring invasive support. Given the retrospective single-center design and the potential for residual confounding, the observed associations should not be interpreted as causal. Further prospective multicenter studies incorporating time-dependent analyses and standardized infection-surveillance definitions are warranted to clarify the independent contribution of HAI to clinical outcomes after aSAH.

## Figures and Tables

**Table 1:** Baseline characteristics, clinical management, and outcomes of the study cohort.

Variable	Study Group (*n* = 106)
Demographics	
Age, years, median (IQR)	54.5 (44.0–65.8)
Female	57 (53.8%)
Male	49 (46.2%)
Body mass index, kg/m^2^, median (IQR)	25.9 (23.4–29.4)
Comorbidities and Risk Factors	
Arterial hypertension	37 (34.9%)
Multiple aneurysm	30 (28.3%)
Diabetes mellitus	13 (12.3%)
Ischemic heart disease	6 (5.7%)
Thyroid disease, overall	7 (6.6%)
Hypothyroidism	6 (5.7%)
Hyperthyroidism	1 (0.9%)
Clinical Status at Admission	
GCS, median (IQR)	13 (5–15)
GCS 3–8	45 (42.5%)
GCS 9–12	7 (6.6%)
GCS 13–15	54 (50.9%)
APACHE II score, median (IQR)	21 (13–27)
WFNS, median (IQR)	4 (2–5)
WFNS I–III	49 (46.2%)
WFNS IV–V	57 (53.8%)
FOUR, median (IQR)	12.5 (7–16)
FOUR 0–12	53 (50.0%)
FOUR 13–16	53 (50.0%)
Fisher score, median (IQR)	4 (3–4)
High Fisher grade	96 (90.6%)
Aneurysm Location	
ACA	7 (6.6%)
ACoA	37 (34.9%)
ICA	13 (12.3%)
MCA	28 (26.4%)
PCoA	5 (4.7%)
VBA/PICA	16 (15.1%)
Treatment Modality	
Endovascular embolization	73 (68.9%)
Surgical clipping	33 (31.1%)
Cerebrospinal fluid drainage	53 (50.0%)
Duration of cerebrospinal fluid drainage time; days	7 (5–8)
EVD	27 (25.5%)
LD	26 (24.5%)
ICH evacuation	20 (18.9%)
Decompressive craniectomy	32 (30.2%)
Intensive Care Respiratory Support	
Mechanical ventilation	91 (85.8%)
Tracheostomy	29 (27.4%)
Time to tracheostomy, days, median (IQR)	11 (10–16)
Duration of mechanical ventilation, days, median (IQR)	8 (2–16.8)
Duration of urinary catheterization, days, median (IQR)	12.5 (7–19)
Duration of Vascular catheter present, days, median (IQR)	10 (6–18)
ICU length of stay, days, median (IQR)	13.5 (8–20)
Total hospital length of stay, days, median (IQR)	19 (14–43.2)
Neurological complications	
DCI (*n* = 101)	61 (60.4%)
Day of DCI onset after ictus, days, median [IQR)	6 (4–8)
TCD vasospasm (*n* = 105)	62 (59.0%)
Hydrocephalus	22 (20.8%)
Seizures	21 (19.8%)
Clinical Outcome at Discharge	
GOS, median (IQR)	3 (1–4)
Favorable outcome, GOS 4–5	40 (37.7%)
Unfavorable outcome, GOS 1–3	66 (62.3%)
In-hospital mortality	31 (29.2%)
Discharge home	41 (38.7%)
Transfer to neurology department in another hospital	13 (12.3%)
Transfer to rehabilitation center	8 (7.5%)
Discharge to a long-term care facility	8 (7.5%)
Discharge to hospice	5 (4.7%)

IQR—interquartile range, GCS—Glasgow Coma Scale, APACHE II—Acute Physiology and Chronic Health Evaluation, WFNS—World Federation of Neurosurgical Societies, FOUR—Full Outline of UnResponsiveness, ACA—Anterior Cerebral Artery, ACoA—Anterior Communicating Artery, ICA—Internal Carotid Artery, MCA—middle cerebral artery, PCoA—Posterior Communicating Artery, VBA—Vertebrobasilar Artery, PICA—Posterior Inferior Cerebellar Artery, EVD—external ventricular drainage, LD—lumbar drainage, ICH—Intracerebral Hemorrhage, ICU—intensive care unit, DCI—delayed cerebral ischemia, TCD—Transcranial Doppler Ultrasonography, GOS—Glasgow Outcome Scale.

**Table 2:** Timing and frequency of healthcare-associated infections.

Variable	*n*	%	Median Onset, Days	IQR, Days	Range, Days
First Infectious Episode Among Patients with HAIs (*n* = 47)					
Overall first infectious episode	47	100	10	8–12	4–29
Days 1–3	0	0.0	—	—	—
Days 4–7	11	23.4	—	—	—
Days 8–14	26	55.3	—	—	—
>14 days	10	21.3	—	—	—
Timing According to Infection Type (*n* = 106)					
VAP	30	28.3	12	10–17	5–31
CAUTI	24	22.6	12	9–19.5	4–35
CSF	13	12.3	12	8–15	4–28
CLABSI	9	8.5	11	10–21	6–36

IQR—interquartile range, HAIs—healthcare-associated infections, VAP—ventilator-associated pneumonia, CAUTI—catheter-associated urinary tract infection, CSF—cerebrospinal fluid infection, CLABSI—central line-associated bloodstream infection. Percentages for individual infection types were calculated using the total study population (*n* = 106) as the denominator. Percentages in the first HAI section were calculated among patients who developed at least one HAI (*n* = 47).

**Table 3:** Distribution of healthcare-associated infections according to year of hospitalization.

Year	Patients, *n*	Any HAI, *n* (%)	VAP, *n* (%)	CAUTI, *n* (%)	CSF Infection, *n* (%)	CLABSI, *n* (%)	Total Recorded HAI Episodes, *n*
2019	26	11 (42.3%)	7 (26.9%)	9 (34.6%)	2 (7.7%)	1 (3.8%)	19
2020	12	5 (41.7%)	4 (33.3%)	2 (16.7%)	2 (16.7%)	2 (16.7%)	10
2021	13	8 (61.5%)	3 (23.1%)	4 (30.8%)	4 (30.8%)	3 (23.1%)	14
2022	17	9 (52.9%)	7 (41.2%)	4 (23.5%)	2 (11.8%)	3 (17.6%)	16
2023	11	5 (45.5%)	4 (36.4%)	1 (9.1%)	1 (9.1%)	0 (0.0%)	6
2024	27	9 (33.3%)	5 (18.5%)	4 (14.8%)	2 (7.4%)	0 (0.0%)	11
Total	106	47 (44.3%)	30 (28.3%)	24 (22.6%)	13 (12.3%)	9 (8.5%)	76

HAI—healthcare-associated infection, VAP—ventilator-associated pneumonia, CAUTI—catheter-associated urinary tract infection, CSF—cerebrospinal fluid infection, CLABSI—central line-associated bloodstream infection.

**Table 4:** Comparison of clinical course and outcomes between patients with a single HAI type and those with multiple HAI types.

Variable	Single HAI Type (*n* = 24)	≥2 HAI Types (*n* = 23)	*p*-Value
Intensive Care Treatment			
Mechanical ventilation	24 (100.0%)	23 (100.0%)	1.000
Duration of mechanical ventilation, days, median (IQR)	13.5 (7.8–15.3)	23 (19–30.5)	<0.001
Tracheostomy	7 (29.2%)	17 (73.9%)	0.003
Time to tracheostomy	12 (7–16)	11 (10–20)	0.482
Duration of urinary catheterization, days, median (IQR)	15.5 (12.8–19)	28 (23–36)	<0.001
Duration of Vascular catheter present, days, median (IQR)	14.5 (10.8–18.3)	24 (18–30)	<0.001
Hospitalization Course			
Total hospital length of stay, days, median (IQR)	23 (15.8–58)	54 (36–62.5)	0.014
ICU length of stay, days, median (IQR)	15.5 (12.8–19)	30 (24–36)	<0.001
Clinical Outcome			
GOS score, median (IQR]	3 (1–3]	2 (1–3]	0.287
Unfavorable outcome, GOS 1–3	20 (83.3%)	21 (91.3%)	0.666
Favorable outcome, GOS 4–5	4 (16.7%)	2 (8.7%)	0.666
In-hospital mortality	9 (37.5%)	7 (30.4%)	0.760
Discharge home	5 (20.8%)	3 (13.0%)	0.701
Transfer to neurology department in another hospital	3 (12.5%)	2 (8.7%)	1.000
Transfer to rehabilitation center	2 (8.3%)	4 (17.4%)	0.416
Discharge to a long-term care facility	5 (20.8%)	2 (8.7%)	0.416
Discharge to hospice	0% (0.0%)	5 (21.7%)	0.022

HAI—healthcare-associated infection, ICU—intensive care unit, DCI—delayed cerebral ischemia, GOS—Glasgow Outcome Scale, IQR—interquartile range. Continuous variables were compared using the Mann–Whitney U test. Categorical variables were compared using the chi-square test or Fisher’s exact test, as appropriate. The classification was based on the number of distinct HAI types experienced during hospitalization: one HAI type versus two or more HAI types.

**Table 5:** Antimicrobial resistance patterns among patients who developed healthcare-associated infections.

Resistance Phenotype /Mechanism	Patients with HAI, *n* (%) (*N* = 47)	Bacterial Species Associated with Resistance
MLSB	20 (42.6%)	*S. epidermidis* (*n* = 11), *S. aureus* (*n* = 7), *S. haemolyticus* (*n* = 5), *S. capitis* (*n* = 1), SP † (*n* = 2)
MRS	19 (40.4%)	*S. epidermidis* (*n* = 12), *S. haemolyticus* (*n* = 6), *S. capitis* (*n* = 1), SKU † (*n* = 2), SP † (*n* = 2)
XDR	10 (21.3%)	*A. baumannii* (*n* = 10)
ESBL	9 (19.1%)	*K. pneumoniae* (*n* = 6), *E. coli* (*n* = 3), *E. cloacae* (*n* = 1), PSPP † (*n* = 1), KES † (*n* = 1), KOX † (*n* = 1)
MRSA	9 (19.1%)	*S. aureus* (*n* = 9)
MDR	8 (17.0%)	*A. baumannii* (*n* = 7), *P. aeruginosa* (*n* = 1), PE † (*n* = 1)
MBL	3 (6.4%)	*K. pneumoniae* (*n* = 2), PE † (*n* = 1)
NDM	3 (6.4%)	*K. pneumoniae* (*n* = 3)
HLAR	2 (4.3%)	*E. faecalis* (*n* = 2)
AmpC	2 (4.3%)	*P. mirabilis* (*n* = 2)
VIM	2 (4.3%)	*K. pneumoniae* (*n* = 1), *P. aeruginosa* (*n* = 1)

HAI—healthcare-associated infection, MLSB—macrolide–lincosamide–streptogramin B, MRS—methicillin-resistant staphylococci, XDR—extensively drug-resistant, ESBL—Extended-Spectrum β-Lactamase, MRSA—methicillin-resistant *Staphylococcus aureus*, MDR—multidrug-resistant bacteria, MBL—Metallo-β-Lactamase, NDM—New Delhi Metallo-β-Lactamase, HLAR—High-Level Aminoglycoside Resistance, AmpC—AmpC β-Lactamase, VIM—Verona Integron-Encoded Metallo-β-Lactamase, SP—*Staphylococcus* spp., SKU—coagulase-negative staphylococci, PSPP—*Proteus*, *Serratia*, *Providencia*, *Proteus* spp., KES—*Klebsiella*–*Enterobacter*–*Serratia* spp., KOX—*Klebsiella oxytoca*, PE—*Pseudomonas* spp. † Laboratory group designation used when the isolate was not identified to species level; expansions are given below.

**Table 6:** Baseline characteristics according to infection status.

Variable	No Infection (*n* = 59)	Infection (*n* = 47)	*p*-Value
Demographic Characteristics			
Age, years, median (IQR)	54 (43–65)	56 (47.5–66)	0.2837
Female	29 (49.2%)	28 (59.6%)	0.3826
Male	30 (50.8%)	19 (40.4%)	
BMI, kg/m^2^, median (IQR)	26 (23.4–29.4)	25.9 (23.4–28.9)	0.6699
Comorbidities			
Arterial hypertension	19 (32.2%)	18 (38.3%)	0.6535
Ischemic heart disease	4 (6.8%)	2 (4.3%)	0.6910
Diabetes mellitus	7 (11.9%)	6 (12.8%)	1.0000
Clinical Severity at Admission			
GCS score, median (IQR)	14 (5.5–15)	8 (5–13)	0.0231
GCS 3–8	17 (28.8%)	28 (59.6%)	0.0017
WFNS score, median (IQR)	2 (1–5)	5 (4–5)	<0.001
WFNS IV–V	21 (35.6%)	36 (76.6%)	<0.001
Fisher score, median (IQR)	4 (3–4)	4 (4–4)	<0.001
High Fisher score	49 (83.1%)	47 (100.0%)	0.0021
APACHE score, median (IQR)	14 (10.5–25)	24 (18.5–29.5)	<0.001

IQR—interquartile range, BMI—body mass index, GCS—Glasgow Coma Scale, WFNS—World Federation of Neurosurgical Societies, APACHE—Acute Physiology and Chronic Health Evaluation.

**Table 7:** Hospital course and clinical outcomes according to infection status.

Variable	No Infection (*n* = 59)	Infection (*n* = 47)	*p*-Value
Intensive Care Treatment			
Mechanical ventilation	44 (74.6%)	47 (100.0%)	<0.001
Duration of mechanical ventilation, days, median (IQR)	3 (1–7.5)	18 (12–24.5)	<0.001
Tracheostomy	5 (8.5%)	24 (51.1%)	<0.001
Time to tracheostomy, days, median (IQR)	10 (10–14)	11.5 (9.8–18.5)	0.8609
Cerebrospinal fluid drainage	23 (39.0%)	30 (63.8%)	0.0190
Duration of cerebrospinal fluid drainage time; days	6 (5–7)	7 (7–9.5)	0.0053
EVD	9 (15.3%)	18 (38.3%)	0.0131
LD	14 (23.7%)	12 (25.5%)	
Duration of urinary catheterization, days, median (IQR)	8 (5–12)	20 (15–30)	<0.001
Duration of Vascular catheter present, days, median (IQR)	7 (5–10)	18 (13–24.5)	<0.001
Hospitalization Course			
Total hospital length of stay, days, median (IQR)	18 (11–19.5)	44 (19–61)	<0.001
ICU length of stay, days, median (IQR)	8 (5–12)	21 (15.5–30)	<0.001
Neurological complications			
DCI	24/55 (43.6%)	37/46 (80.4%)	<0.001
Day of DCI onset after ictus, days, median (IQR)	5 (4–7)	7 (5–9)	0.1114
TCD vasospasm	26/58 (44.8%)	36/47 (76.6%)	0.0020
Hydrocephalus	7 (11.9%)	15 (31.9%)	0.0222
Seizures	8 (13.6%)	13 (27.7%)	0.1178
Clinical Outcome			
GOS score, median (IQR)	4 (1–5)	2 (1–3)	0.0005
Unfavorable outcome, GOS 1–3	25 (42.4%)	41 (87.2%)	<0.001
Favorable outcome, GOS 4–5	34 (57.6%)	6 (12.8%)	<0.001
In-hospital mortality	15 (25.4%)	16 (34.0%)	0.4507
Discharge home	33 (55.9%)	8 (17.0%)	<0.001
Transfer to neurology ward department in another hospital	8 (13.6%)	5 (10.6%)	0.875
Transfer to rehabilitation center	2 (3.4%)	6 (12.8%)	0.135
Discharge to a long-term care facility	1 (1.7%)	7 (14.9%)	0.021
Discharge to hospice	0 (0.0%)	5 (10.6%)	0.015

IQR—interquartile range, ICU—intensive care unit, GOS—Glasgow Outcome Scale.

**Table 8:** Temporal relationship between delayed cerebral ischemia and healthcare-associated infection.

Infection Type	Before DCI	After DCI	Total, *n*
VAP	5	20	25
CAUTI	4	17	21
CLABSI	1	7	8
CSF infection	1	7	8
Total	11 (17%)	51(82%)	62

DCI—delayed cerebral ischemia, CLABSI—central line-associated bloodstream infection, CSF—cerebrospinal fluid infection, VAP—ventilator-associated pneumonia, CAUTI—catheter-associated urinary tract infection.

**Table 9:** Multivariable cause-specific Cox regression analysis of factors associated with the first healthcare-associated infection.

Variable	HR	95% CI	*p*-Value
Age, per 1-year increase	1.005	0.985–1.026	0.632
Male sex	1.126	0.596–2.128	0.715
GCS	0.921	0.865–0.981	0.011
FOUR	0.889	0.832–0.951	<0.001
APACHE II	1.072	1.034–1.112	<0.001
High WFNS grade (IV–V)	4.168	2.072–8.384	<0.001
Fisher	2.886	1.237–6.735	0.014

HR—hazard ratio; CI—confidence interval; HAI—healthcare-associated infection; WFNS—World Federation of Neurosurgical Societies. Time to first HAI was analyzed using a cause-specific Cox proportional hazards model. Death and discharge without HAI were treated as competing events. The primary model included age, sex, and high WFNS grade. Because severity scales are strongly intercorrelated, GCS, FOUR, and APACHE II were each entered in place of WFNS grade in separate sensitivity models adjusted for age and sex; Fisher grade was added to the primary model in a further sensitivity analysis. Each row therefore reports the estimate from the model in which that variable was included. All models comprised 106 patients and 47 first HAI events. The proportional hazards assumption was satisfied (global Schoenfeld test, *p* = 0.93).

**Table 10:** Multivariable time-to-event analyses of in-hospital mortality and live hospital discharge.

Variable	In-Hospital Mortality		Live Hospital Discharge	
	HR (95% CI)	*p*-Value	HR (95% CI)	*p*-Value
HAI, time-dependent	1.178 (0.478–2.902)	0.721	0.295 (0.164–0.529)	<0.001
Age, per 1-year increase	1.037 (1.009–1.066)	0.009	0.983 (0.966–1.000)	0.055
High WFNS grade (IV–V)	2.169 (0.891–5.278)	0.088	0.538 (0.326–0.889)	0.016

HR—hazard ratio; CI—confidence interval; HAI—healthcare-associated infection; WFNS—World Federation of Neurosurgical Societies. HAI was modeled as a time-dependent exposure. In-hospital mortality was analyzed using Cox regression adjusted for age and WFNS grade (*N* = 106; 31 deaths). Live hospital discharge was analyzed using a cause-specific Cox model with death treated as a competing event (*N* = 106; 75 live discharges). For the live-discharge model, HR < 1 indicates a lower instantaneous hazard of live hospital discharge.

**Table 11:** Partial proportional odds regression analysis of Glasgow Outcome Scale at hospital discharge.

Variable	OR	95% CI	*p*-Value
HAI: GOS ≤ 1 vs. > 1	0.715	0.264–1.933	0.508
HAI: GOS ≤ 2 vs. > 2	1.477	0.565–3.860	0.426
HAI: GOS ≤ 3 vs. > 3	7.939	2.580–24.436	<0.001
HAI: GOS ≤ 4 vs. > 4	5.668	1.167–27.521	0.031
Age, per 1-year increase	1.069	1.039–1.101	<0.001
Male sex	1.571	0.728–3.391	0.250
High WFNS grade (IV–V)	6.593	2.743–15.844	<0.001

OR—odds ratio; CI—confidence interval; HAI—healthcare-associated infection; GOS—Glasgow Outcome Scale; WFNS—World Federation of Neurosurgical Societies. Because the proportional-odds assumption was violated (Brant omnibus *p* = 0.003), a partial proportional odds model was used. The effect of HAI was allowed to vary across GOS thresholds, whereas age, sex, and WFNS grade were constrained to be proportional. For threshold-specific HAI effects, OR > 1 indicates greater odds of being at or below the stated GOS threshold. The analysis included 106 patients.

## Data Availability

The data presented in this study are not publicly available due to ethical and privacy restrictions, as they contain potentially identifiable clinical information. Anonymized data may be made available from the corresponding author upon request.

## Supplementary Materials

The following supporting information can be downloaded at https://www.mdpi.com/article/10.3390/jcm15176718/s1, Table S1: Epidemiological Measures of Healthcare-Associated Infections, Table S2: Microorganisms Isolated from Qualifying Clinical Specimens in Patients with Healthcare-Associated Infections.

## Abbreviations

The following abbreviations are used in this manuscript:

ACA	Anterior Cerebral Artery
ACoA	Anterior Communicating Artery
AmpC	AmpC β-Lactamase
APACHE II	Acute Physiology and Chronic Health Evaluation II
aSAH	Aneurysmal Subarachnoid Hemorrhage
BSI	Bloodstream Infection
BMI	Body Mass Index
CAUTI	Catheter-Associated Urinary Tract Infection
CIDS	Central Nervous System Injury-Induced Immunodepression
CI	Confidence Interval
CLABSI	Central Line-Associated Bloodstream Infection
CNS	Central Nervous System
COVID-19	Coronavirus Disease 2019
CRP	C-reactive Protein
CSF	Cerebrospinal Fluid
CT	Computed Tomography
CTA	Computed Tomography Angiography
CVC	Central Venous Catheter
DAMPs	Damage-Associated Molecular Patterns
DCI	Delayed Cerebral Ischemia
DSA	Digital Subtraction Angiography
EBI	Early Brain Injury
ECDC	European Centre for Disease Prevention and Control
ENIRRIs	European Network for ICU-Related Respiratory Infections
ESBL	Extended-Spectrum β-Lactamase
EUCAST	European Committee on Antimicrobial Susceptibility Testing
EVD	External Ventricular Drainage
FOUR	Full Outline of UnResponsiveness
GCS	Glasgow Coma Scale
GOS	Glasgow Outcome Scale
GRE	Glycopeptide-Resistant Enterococci
HAI	Healthcare-Associated Infection
HAP	Hospital-Acquired Pneumonia
HLAR	High-Level Aminoglycoside Resistance
HPA	Hypothalamic–Pituitary–Adrenal
HR	Hazard Ratio
ICA	Internal Carotid Artery
ICU	Intensive Care Unit
ICH	Intracerebral Hemorrhage
IL-1β	Interleukin-1 beta
IL-6	Interleukin-6
IQR	Interquartile Range
IRR	Incidence Rate Ratio
LD	Lumbar Drainage
MBL	Metallo-β-Lactamase
MCA	Middle Cerebral Artery
MDR	Multidrug-Resistant
MLSB	Macrolide–Lincosamide–Streptogramin B
MRI	Magnetic Resonance Imaging
MRS	Methicillin-Resistant Staphylococci
MRSA	Methicillin-Resistant *Staphylococcus aureus*
NDM	New Delhi Metallo-β-Lactamase
NRS-2002	Nutritional Risk Screening 2002
OR	Odds Ratio
PCoA	Posterior Communicating Artery
PCR	Polymerase Chain Reaction
PCT	Procalcitonin
PE	*Pseudomonas* spp.
PICA	Posterior Inferior Cerebellar Artery
RASS	Richmond Agitation-Sedation Scale
SAH	Subarachnoid Hemorrhage
SIRS	Systemic Inflammatory Response Syndrome
SP	*Staphylococcus* spp.
spp.	Species pluralis
STROBE	Strengthening the Reporting of Observational Studies in Epidemiology
TCD	Transcranial Doppler
TNF-α	Tumor Necrosis Factor-alpha
VAP	Ventilator-Associated Pneumonia
VBA	Vertebrobasilar Artery
VIM	Verona Integron-Encoded Metallo-β-Lactamase
WFNS	World Federation of Neurosurgical Societies
XDR	Extensively Drug-Resistant
