# Supplementary material for: Healthcare-Associated Infections After Aneurysmal Subarachnoid Hemorrhage: A Retrospective Single-Center Cohort Study
Source: J Clin Med. 2026 Aug 29;15(17):6718. doi: 10.3390/jcm15176718 (PMC13565980; doi:10.3390/jcm15176718)
Supplement: Supplementary file 1 [file jcm-15-06718-s001.zip › jcm-4468545-supplementary.pdf]

Supplementary Table S1. Epidemiological Measures of Healthcare-Associated Infections

Frequency, patient-day incidence rates, device-normalized incidence densities, and device utilization ratios in the aSAH cohort (N = 106). All values were derived from the final adjudicated analytic dataset.

A. Infection frequency, patient-day incidence rates, and device-normalized incidence densities

Indicator	Formula	Reporting unit	Numerator	Denominator	Study value (95% CI)
Infection frequency					
Patients with ≥1 HAI	Patients with ≥1 HAI / total patients × 100	%	47	106	44.3% (35.2–53.8)
VAP frequency	VAP episodes / total patients × 100	%	30	106	28.3% (20.6–37.5)
CAUTI frequency	CAUTI episodes / total patients × 100	%	24	106	22.6% (15.7–31.5)
CLABSI frequency	CLABSI episodes / total patients × 100	%	9	106	8.5% (4.5–15.4)
CSF infection frequency	CSF infection episodes / total patients × 100	%	13	106	12.3% (7.3–19.9)
Patient-day incidence rates					
Overall HAI incidence density – hospital	All HAI episodes / hospital patient-days × 1,000	per 1,000 hospital patient-days	76	3,507	21.7 (17.1–27.1)
Overall HAI incidence density – ICU	All HAI episodes / ICU patient-days × 1,000	per 1,000 ICU patient-days	76	1,650	46.1 (36.3–57.7)
VAP incidence rate	VAP episodes / hospital patient-days × 1,000	per 1,000 hospital patient-days	30	3,507	8.6 (5.8–12.2)
CAUTI incidence rate	CAUTI episodes / hospital patient-days × 1,000	per 1,000 hospital patient-days	24	3,507	6.8 (4.4–10.2)
CLABSI incidence rate	CLABSI episodes / hospital patient-days × 1,000	per 1,000 hospital patient-days	9	3,507	2.6 (1.2–4.9)
CSF infection incidence rate	CSF infection episodes / hospital patient-days × 1,000	per 1,000 hospital patient-days	13	3,507	3.7 (2.0–6.3)
Device-normalized incidence densities					
VAP incidence density	VAP episodes / mechanical ventilation-days × 1,000	per 1,000 MV-days	30	1,145	26.2 (17.7–37.4)
CAUTI incidence density	CAUTI episodes / urinary catheter-days × 1,000	per 1,000 catheter-days	24	1,623	14.8 (9.5–22.0)
CLABSI incidence density	CLABSI episodes / central venous catheter-days × 1,000	per 1,000 CVC-days	9	1,371	6.6 (3.0–12.5)
CSF infection rate normalized to EVD-days	CSF infection episodes in EVD-exposed patients / EVD-days × 1,000	per 1,000 EVD-days	7	214	32.7 (13.2–67.4)

Indicator	Formula	Reporting unit	Numerator	Denominator	Study value (95% CI)
CSF infection rate normalized to LD-days	CSF infection episodes in LD-exposed patients / LD-days × 1,000	per 1,000 LD-days	4	159	25.2 (6.9–64.4)

Notes. Proportions are presented with Wilson 95% confidence intervals; incidence rates and incidence densities are presented with exact Poisson 95% confidence intervals. The cohort contributed 3,507 total hospital patient-days and 1,650 ICU patient-days. Site-specific counts denote affected patients, as no patient had more than one episode at the same infection site.

Device association was adjudicated for every episode against the recorded device-exposure period, applying a single prespecified rule in accordance with the ECDC HAI-Net ICU protocol, version 2.2: an infection was classified as device-associated when the relevant device was in place at any point during the 48 hours preceding the onset of infection, and within the seven days preceding onset for catheter-associated urinary tract infection. Where a device had been removed and subsequently reinserted, each period of device exposure was recorded separately, and device association was determined with reference to the most recent period of exposure preceding the infection. Device-days were calculated as the total number of calendar days during which the corresponding device was in place, summed across all periods of device exposure. All VAP, CAUTI and CLABSI episodes fulfilled these criteria and contributed to the corresponding device-day-normalized rates presented here.

All pneumonia episodes occurred in patients in whom invasive mechanical ventilation was in place within the 48 hours preceding infection onset and were classified as ventilator-associated pneumonia (VAP), including four episodes occurring after reintubation following a period without invasive ventilatory support; no hospital-acquired pneumonia occurring independently of invasive mechanical ventilation was identified. All bloodstream infection episodes fulfilled the predefined central line-association criteria and were classified as central line-associated bloodstream infection (CLABSI), including two episodes occurring after reinsertion of a central venous catheter following a catheter-free interval. All urinary tract infections occurred while an indwelling urinary catheter was in situ and met the predefined catheter-associated urinary tract infection (CAUTI) criteria.

Rates of CSF infection normalized to EVD-days and lumbar drain-days describe the occurrence of CSF infection during periods of documented drain exposure and should not be interpreted as formal EVD-associated or lumbar drain-associated infection rates, as two CSF infection episodes occurred in patients without recorded drainage exposure.

HAI = healthcare-associated infection; VAP = ventilator-associated pneumonia; CAUTI = catheter-associated urinary tract infection; CLABSI = central line-associated bloodstream infection; CSF = cerebrospinal fluid; EVD = external ventricular drain; LD = lumbar drain; MV = mechanical ventilation; CVC = central venous catheter; ICU = intensive care unit.

B. Device utilization ratios

Indicator	Formula	Reporting unit	Numerator	Denominator	Study value
Mechanical ventilation utilization ratio (MV-UR)	Mechanical ventilation-days / ICU patient-days × 100	%	1,145	1,650	69.4%
Urinary catheter utilization ratio (UC-UR)	Urinary catheter-days / ICU patient-days × 100	%	1,623	1,650	98.4%
Central venous catheter utilization ratio (CVC-UR)	Central venous catheter-days / ICU patient-days × 100	%	1,371	1,650	83.1%
EVD utilization ratio (EVD-UR)	EVD-days / ICU patient-days × 100	%	214	1,650	13.0%
LD utilization ratio (LD-UR)	LD-days / ICU patient-days × 100	%	159	1,650	9.6%

Note. Device utilization ratio = device-days / total ICU patient-days × 100. The denominator for all utilization ratios was 1,650 ICU patient-days. Device-days were summed across all periods of device exposure, including repeated periods of exposure in patients who underwent device removal and subsequent reinsertion. EVD = external ventricular drain; ICU = intensive care unit; LD = lumbar drain.

Supplementary Table S2. Microorganisms Isolated from Qualifying Clinical Specimens in Patients with Healthcare-Associated Infections

Generated from the final adjudicated analytic dataset (N = 106 patients; 76 infection episodes).

**Infection type**	**Microorganism**	***n***	**%**
Ventilator-associated pneumonia (n = 30)	*Acinetobacter baumannii*	17	56.7
	Staphylococcus aureus	8	26.7
	Klebsiella pneumoniae	7	23.3
	Streptococcus oralis	7	23.3
	Escherichia coli	5	16.7
	Pseudomonas aeruginosa	5	16.7
	Enterococcus faecalis	4	13.3
	Candida albicans	3	10.0
	Candida glabrata	3	10.0
	Candida krusei	3	10.0
	Coagulase-negative staphylococci	3	10.0
	Corynebacterium spp.	3	10.0
	Serratia marcescens	3	10.0
	Candida spp.	2	6.7
	Enterobacter cloacae	2	6.7
	Proteus mirabilis	2	6.7
	Candida dubliniensis	1	3.3
	Candida inconspicua	1	3.3
	Candida lusitaniae	1	3.3
	Candida tropicalis	1	3.3
	Enterococcus faecium	1	3.3
	Klebsiella oxytoca	1	3.3
	Stenotrophomonas maltophilia	1	3.3
	Gram-negative bacillus, not further identified	1	3.3
Catheter-associated urinary tract infection (n = 24)	*Enterococcus faecalis*	5	20.8
	Candida glabrata	4	16.7
	Acinetobacter baumannii	3	12.5
	Candida albicans	3	12.5
	Proteus mirabilis	3	12.5
	Klebsiella pneumoniae	2	8.3
	Candida parapsilosis	1	4.2
	Candida spp.	1	4.2
	Enterococcus faecium	1	4.2
	Escherichia coli	1	4.2
	Geotrichum spp.	1	4.2
	Klebsiella oxytoca	1	4.2
	Staphylococcus epidermidis	1	4.2
Central line-associated bloodstream infection (n = 9)	*Acinetobacter baumannii*	4	44.4
	Staphylococcus epidermidis	4	44.4
	Staphylococcus haemolyticus	2	22.2
	Acinetobacter pittii	1	11.1
	Bacillus cereus	1	11.1

Infection type	Microorganism	*n*	%
	Candida spp.	1	11.1
	Escherichia coli	1	11.1
	Klebsiella pneumoniae	1	11.1
	Staphylococcus pettenkoferi	1	11.1
	Gram-negative bacillus, not further identified	1	11.1
Cerebrospinal fluid infection (n = 13)	*Staphylococcus epidermidis*	4	30.8
	Acinetobacter baumannii	3	23.1
	Bacillus subtilis	1	7.7
	Enterococcus faecalis	1	7.7
	Escherichia coli	1	7.7
	Klebsiella pneumoniae	1	7.7
	Proteus mirabilis	1	7.7
	Staphylococcus capitis	1	7.7
	Staphylococcus haemolyticus	1	7.7

Notes. Counts denote the number of patients from whom the microorganism was recovered. Percentages were calculated within each infection category against denominators of 30 ventilator-associated pneumonia, 24 catheter-associated urinary tract infection, 9 central line-associated bloodstream infection and 13 cerebrospinal fluid infection episodes. Because more than one microorganism could be recovered from a single episode, percentages may sum to more than 100% within a category. Infection categories and device-association criteria correspond to those applied throughout the Methods, Results and Supplementary Table S1. Isolation of a microorganism from a qualifying specimen is not in itself evidence of its etiological role.

Two isolates were documented only as Gram-negative bacilli, without identification to genus or species level: one recovered from a ventilator-associated pneumonia episode and one from a central line-associated bloodstream infection episode. These were two separate isolates, and both are listed here as unidentified Gram-negative bacilli. The organism-specific profile shown in Figure 6 comprises only those central line-associated bloodstream infection isolates identified to species level, whereas the present table lists all microorganisms recovered; percentages in both are calculated from all nine episodes.

Microbiological confirmation was not obtained in two cerebrospinal fluid infection episodes; both fulfilled the predefined clinical and cerebrospinal fluid-based criteria specified in Section 2.5 and are retained in the denominator of 13. Microorganism nomenclature is reported as recorded by the clinical microbiology laboratory during the study period.
